# Publisher Correction: Multi-resolution localization of causal variants across the genome

**DOI:** 10.1038/s41467-020-15690-2

**Published:** 2020-04-07

**Authors:** Matteo Sesia, Eugene Katsevich, Stephen Bates, Emmanuel Candès, Chiara Sabatti

**Affiliations:** 1 Department of Statistics, Stanford University, Stanford, CA 94305 USA; 2 Department of Statistics, Carnegie Mellon University, Pittsburgh, PA 15213 USA; 3 Departments of Mathematics and of Statistics, Stanford University, Stanford, CA 94305 USA; 4 Departments of Biomedical Data Science and of Statistics, Stanford University, Stanford, CA 94305 USA

**Keywords:** Statistical methods, Genome-wide association studies, Genetic variation, Haplotypes

Correction to: *Nature Communications* 10.1038/s41467-020-14791-2, published online 27 February 2020.

The original version of this Article contained an error in the author affiliations.

The affiliation of Matteo Sesia and Stephen Bates with Department of Statistics, Stanford University, Stanford, CA, 94305, USA was inadvertently omitted.

This has now been corrected in both the PDF and HTML versions of the Article.

