# Supplementary material for: Multi-resolution localization of causal variants across the genome
Source: Nat Commun. 2020 Feb 27;11:1093. doi: 10.1038/s41467-020-14791-2 (PMC7046731; doi:10.1038/s41467-020-14791-2)
Supplement: Supplementary file 2 — Supplementary Information [file 41467_2020_14791_MOESM2_ESM.pdf]

Supplementary Information for
Multi-resolution localization of causal variants across the genome

Matteo Sesia, Eugene Katsevich, Stephen Bates, Emmanuel Candès,* Chiara Sabatti*

Stanford University, Department of Statistics, Stanford, CA 94305, USA

* Corresponding authors.

SUPPLEMENTARY METHODS

Knockoffs for composite conditional hypotheses. A typical GWAS involves so many variants in LD that the conditional importance of a single SNP given all others may be very difficult to resolve. We simplify this problem by clustering the loci into LD blocks and testing group-wise conditional hypotheses (Methods). In particular, we test whether all variants in any given block are independent of the trait conditional on the rest of the genome.

Consider the following explanatory example. A trait Y is described by a linear model (for simplicity) with 4 explanatory variables: $Y = \sum_{j=1}^{4} X_j \beta_j + \epsilon$ and Gaussian noise ϵ. Given independent observations of $\mathbf{X}$ and Y, drawn with $\beta_1 = 1$ and $\beta_j = 0$ for all $j \neq 1$, we want to discover which variables influence Y (i.e., X_1). To make this example interesting, we imagine an extreme form of LD: $X_1 = X_2$ and $X_3 = X_4$, while X_1 and X_3 are independent. This makes it impossible to retrospectively understand whether it is X_1 or X_2 that affects Y. However, we can still hope to conclude that either X_1 or X_2 are important. In fact, introductory statistics classes teach us how to do this with an F-test, as opposed to a t-test, which would have no power in this case.

To solve the above problem within our framework (Methods), we begin by generating powerful knockoffs specifically designed to test group-wise conditional hypotheses. The exact structure of the LD blocks must be taken into account when we create knockoffs. For example, if we naïvely generate knockoffs $\tilde{\mathbf{X}} = (\tilde{X}_1, \tilde{X}_2, \tilde{X}_3, \tilde{X}_4)$ that are pairwise exchangeable with $\mathbf{X}$ one-by-one, we must set $\tilde{X}_1 = X_2 = X_1$ to preserve the equality in distribution between $(\tilde{X}_1, X_2, X_3, X_4)$ and $(X_1, \tilde{X}_2, \tilde{X}_3, \tilde{X}_4)$ when $\tilde{X}_1$ is swapped with X_1. Furthermore, by the same argument we conclude that we need $\tilde{\mathbf{X}} = \mathbf{X}$. However, these knockoffs are powerless as negative controls (although they are exchangeable). Fortunately, we can generate powerful group-knockoffs (Methods) under milder exchangeability constraints that can still be used to test grouped hypotheses. In the above toy example, we consider the partition $\mathcal{G} = (\{1,2\},\{3,4\})$, and require that $\tilde{X}_1 = \tilde{X}_2$ and $\tilde{X}_3 = \tilde{X}_4$, while allowing $\tilde{\mathbf{X}} \neq \mathbf{X}$.

Definition 1 (Group-knockoffs)**.** *Consider random variables* $\tilde{\boldsymbol{Z}} = (Z_1, \dots, Z_p)$*, and a partition* $\mathcal{G} = (G_1, \dots, G_L)$ *of* $\{1, \dots, p\}$*. Then* $\tilde{\boldsymbol{Z}} = (\tilde{Z}_1, \dots, \tilde{Z}_p)$ *is said to be a* group-knockoff *for* $\tilde{\boldsymbol{Z}}$ *with respect to* $\mathcal{G}$ *if for each group* $G \in \mathcal{G}$*, we have:*

$$(\boldsymbol{Z}, \tilde{\boldsymbol{Z}})_{\text{swap}(G;\mathcal{G})} \stackrel{d}{=} (\boldsymbol{Z}, \tilde{\boldsymbol{Z}}). \tag{1}$$

Above, $(\boldsymbol{Z}, \tilde{\boldsymbol{Z}})_{\text{swap}(G;\mathcal{G})}$ *means that the jth coordinate is swapped with the* $(j+p)$*th coordinate* $\forall j \in G$.

Algorithms for knockoff generation. The construction of knockoffs for groups of genetic variants is the heart of the methodology in this paper and requires a significant extension of the existing algorithms.[2] Our contribution has two main components: first, we construct group-knockoffs for Markov chains and HMMs; second, we specialize these ideas to obtain fast algorithms for the models of interest in a GWAS. The details of these contributions are here, while the associated technical proofs are at the end of this section.

We begin with group-knockoffs for Markov chains.[2] We say that $\mathbf{Z} = (Z_1, \ldots, Z_p)$, with each variable taking values in $\{1, \ldots, K\}$ for some $K \in \mathbb{N}$, is a discrete Markov chain if its joint probability mass function can be written as:

$$\mathbb{P}[Z_1 = z_1, \ldots, Z_p = z_p] = Q_1(z_1) \prod_{j=2}^{p} Q_j(z_j \mid z_{j-1}). \tag{2}$$

Above, $Q_1(z_1) = \mathbb{P}[Z_1 = z_1]$ denotes the initial distribution of the chain, while the transition matrices between consecutive variables are: $Q_j(z_j \mid z_{j-1}) = \mathbb{P}[Z_j = z_j \mid Z_{j-1} = z_{j-1}]$.

Since Markov chains have a well-defined sequential structure, the special class of contiguous partitions is of particular interest for generating group-knockoffs.

Definition 2 (Contiguous partition)**.** *For any fixed positive integers $L \leq p$, we call a collection of L sets $\mathcal{G} = (G_1, \ldots, G_L)$ a* contiguous partition *of $\{1, \ldots, p\}$ if $\mathcal{G}$ is a partition of $\{1, \ldots, p\}$ and for any distinct $G, G' \in \mathcal{G}$, either $j < l$ for all $j \in G$ and $l \in G'$ or $j > l$ for all $j \in G$ and $l \in G'$.*

For example, the partition $\mathcal{G} = (G_1, \ldots, G_4)$ of $\{1, \ldots, 10\}$ shown on the left-hand-side of the example below is contiguous, while $\mathcal{G}' = (G'_1, \ldots, G'_3)$, on the right-hand-side, is not.

$$\underbrace{1,2,3}_{G_1}, \underbrace{4,5}_{G_2}, \underbrace{6,7}_{G_3}, \underbrace{8,9,10}_{G_4}, \qquad\qquad \underbrace{1,2,3}_{G'_1}, \underbrace{4,5}_{G'_2}, \underbrace{6,7}_{G'_3}, \underbrace{8,9,10}_{G'_2}.$$

We consider only contiguous partitions when we construct knockoff copies of a Markov chain; if a given partition is not contiguous, we first refine it by splitting all non-contiguous groups.

To simplify the notation in the upcoming result, for any $g \in \{1, \ldots, L\}$ and associated group $G_g \in \mathcal{G}$, we indicate the variables in G_g as: $\mathbf{Z}^g = (Z^g_1, \ldots, Z^g_{m_g}) = (Z_j)_{j \in G_g}$. Similarly, we denote the sequence of transition matrices corresponding to the m_g variables contained in the g-th group by: $Q^g = (Q^g_1, \ldots, Q^g_{m_g}) = (Q_j)_{j \in G_g}$. We set $Q^1_1(k \mid l) = Q_1(k)$ and $Q^{L+1}_1(k \mid l) = 1$, $\forall k, l \leq K$.

Proposition 1. *Let $\mathcal{G} = (G_1, \ldots, G_L)$ be a contiguous partition of $\{1, \ldots, p\}$, such that the g-th group G_g has m_g elements. Suppose that $\boldsymbol{Z}$ is distributed as the Markov chain in (2), with known parameters Q. Then, a group-knockoff copy $\tilde{\boldsymbol{Z}}$, with respect to $\mathcal{G}$, can be obtained by sequentially sampling, for $g = 1, \ldots, L$, the g-th group-knockoff copy $\tilde{\boldsymbol{Z}}^g = (\tilde{Z}_1^g, \ldots, \tilde{Z}_{m_g}^g)$ from:*

$$
\begin{aligned}
p(\boldsymbol{Z}^g \mid \boldsymbol{Z}^{-g}, \tilde{\boldsymbol{Z}}^{1:(g-1)}) = \frac{1}{\mathcal{N}_g(\tilde{Z}_1^{g+1})} \times \frac{Q_1^g(Z_1^g \mid \tilde{Z}_{m_{g-1}}^{g-1})^{\mathbb{1}[g \neq 1]}\, Q_1^g(Z_1^g \mid Z_{m_{g-1}}^{g-1})}{\mathcal{N}_{g-1}(Z_1^g)} \\
\times \left[\prod_{j=2}^{m_g} Q_j^g(Z_j^g \mid Z_{j-1}^g) \right] \times Q_1^{g+1}(Z_1^{g+1} \mid Z_{m_g}^g).
\end{aligned}
\tag{3}
$$

The functions $\mathcal{N}$ are defined recursively as:

$$
\mathcal{N}_g(k) = \sum_{z_1^g, \ldots, z_{m_g}^g} \frac{Q_1^g(Z_1^g \mid \tilde{Z}_{m_{g-1}}^{g-1})^{\mathbb{1}[g \neq 1]}\, Q_1^g(Z_1^g \mid Z_{m_{g-1}}^{g-1})}{\mathcal{N}_{g-1}(Z_1^g)} \times \left[\prod_{j=2}^{m_g} Q_j^g(z_j^g \mid z_{j-1}^g) \right] \times Q_1^{g+1}(k \mid z_{m_g}^g),
\tag{4}
$$

with the convention that $\mathcal{N}_0(z) = 1$ for all z. Therefore, Algorithm 1 is an exact procedure for sampling group-knockoff copies of a Markov chain.

Algorithm 1 Group-knockoffs for a Markov chain

for $g = 1$ to $g = L$ **do**
  for $k = 1$ to $k = K$ **do**
    Compute $\mathcal{N}_g(k)$ according to (4).
  Sample $\tilde{\mathbf{Z}}^g$ according to (3).

Algorithm 1 reduces to the known result for ungrouped knockoffs[2] if $L = p$. In the general case, we can sample from the distribution in (3) as follows. From (3), we can write:

$$
p(\tilde{\mathbf{Z}}^g \mid \mathbf{Z}^{-g}, \tilde{\mathbf{Z}}^{1:(g-1)}) = Q_1^{\star g}(\tilde{Z}_1^g) \prod_{j=2}^{m_g} Q_j^{\star g}(\tilde{Z}_j^g \mid \tilde{Z}_{j-1}^g),
$$

for some initial distribution $Q_1^{\star g}$ and suitable transition matrices $Q_j^{\star g}$. Therefore, $\tilde{\mathbf{Z}}^g$ is conditionally a Markov chain. Assuming for simplicity that $g > 1$, we see from (3) that $Q_1^{\star g}$ is given by:

$$
\begin{aligned}
Q_1^{\star g}(\tilde{z}_1^g) = \frac{1}{\mathcal{N}_g(Z_1^{g+1})} \times \frac{Q_1^g(\tilde{z}_1^g \mid \tilde{Z}_{m_{g-1}}^{g-1})\, Q_1^g(\tilde{z}_1^g \mid Z_{m_{g-1}}^{g-1})}{\mathcal{N}_{g-1}(\tilde{z}_1^g)} \\
\times \left(\sum_{\tilde{z}_2^g, \ldots, \tilde{z}_{m_g}^g} \left[\prod_{j=2}^{m_g} Q_j^g(\tilde{z}_j^g \mid \tilde{z}_{j-1}^g) \right] \times Q_1^{g+1}(Z_1^{g+1} \mid \tilde{z}_{m_g}^g) \right),
\end{aligned}
$$

while, for $j \in \{2, \dots, m_g\}$,

$$Q_j^{\star g}(\tilde{z}_j^g \mid \tilde{z}_{j-1}^g) = Q_j^g(\tilde{z}_j^g \mid \tilde{z}_{j-1}^g) \times \left(\sum_{\tilde{z}_{j+1}^g, \dots, \tilde{z}_{m_g}^g} \left[\prod_{j'=j+1}^{m_g} Q_{j'}^g(\tilde{z}_{j'}^g \mid \tilde{z}_{j'-1}^g) \right] \times Q_1^{g+1}(Z_1^{g+1} \mid \tilde{z}_{m_g}^g) \right).$$

It is easy to verify that the above quantities can be computed through $m_g - 1$ multiplications of $K \times K$ matrices. Similarly, the functions in (4) can also be computed efficiently. Therefore, the cost of sampling the g-th group-knockoff is $\mathcal{O}(m_g K^3)$, if $m_g > 1$, and $\mathcal{O}(m_g K^2)$, otherwise. The worst-case complexity of Algorithm 1 is $\mathcal{O}(pK^3)$ because $\sum_{g=1}^{L} m_g = p$. Later, we will derive a more efficient implementation in the special case of genetic variables.

We leverage the result in Proposition 1 to derive a construction of group-knockoffs for HMMs, similarly to previous work.[2] We say that $\mathbf{X} = (X_1, \dots, X_p)$, with each variable taking values in a finite state space $\mathcal{X}$, is distributed as an HMM with K hidden states if there exists a vector of latent random variables $\mathbf{Z} = (Z_1, \dots, Z_p)$, with $Z_j \in \{1, \dots, K\}$, such that:

$$\begin{cases} \mathbf{Z} \sim \mathrm{MC}\,(Q) & \text{(latent discrete Markov chain)}, \\ X_j \mid \mathbf{Z} \sim X_j \mid Z_j \overset{\text{ind.}}{\sim} f_j(X_j \mid Z_j) & \text{(emission distribution)}. \end{cases} \tag{5}$$

Above, $\mathrm{MC}\,(Q)$ indicates the law of a discrete Markov chain, as in (2).

Proposition 2. *Suppose $\boldsymbol{X} = (X_1, \dots, X_p)$ is distributed as the HMM in* (5)*, with an associated latent Markov chain $\boldsymbol{Z} = (Z_1, \dots, Z_p)$. Let $\mathcal{G} = (G_1, \dots, G_L)$ be a contiguous partition of $\{1, \dots, p\}$. Then, Algorithm 2 generates $(\tilde{\boldsymbol{X}}, \tilde{\boldsymbol{Z}})$ such that:*

$$\left((\boldsymbol{X}, \tilde{\boldsymbol{X}})_{\mathrm{swap}(G;\mathcal{G})}, (\boldsymbol{Z}, \tilde{\boldsymbol{Z}})_{\mathrm{swap}(G;\mathcal{G})} \right) \overset{d}{=} \left((\boldsymbol{X}, \tilde{\boldsymbol{X}}), (\boldsymbol{Z}, \tilde{\boldsymbol{Z}}) \right), \qquad \forall G \in \mathcal{G}. \tag{6}$$

In particular, this implies that $\tilde{\boldsymbol{X}}$ is a group-knockoff copy of $\boldsymbol{X}$.

Algorithm 2 Group-knockoffs for an HMM

(1) Sample $\mathbf{Z} = (Z_1, \dots, Z_p)$ from $\mathbb{P}[\mathbf{Z} \mid \mathbf{X} = \mathbf{x}]$ using forward-backward sampling.[2]

(2) Sample a group-knockoff copy $\tilde{\mathbf{Z}}$ of $\mathbf{Z}$, with respect to $\mathcal{G}$, using Algorithm 1.

(3) Sample $\tilde{\mathbf{X}}$ from $\mathbb{P}[\mathbf{X} \mid \mathbf{Z} = \tilde{\mathbf{z}}]$. This is trivial by the conditional independence in (5).

The computational complexity of the first step of Algorithm 2 is known to be $\mathcal{O}(pK^2)$,[2] while the worst-case cost of the second step is $\mathcal{O}(pK^3)$. The complexity of the third step is $\mathcal{O}(p|\mathcal{X}|)$. Therefore, the worst-case total complexity of Algorithm 2 is $\mathcal{O}(p(K^3 + |\mathcal{X}|))$. Later, we will reduce this in the special case of the fastPHASE HMM by simplifying the first two steps analytically.

Knockoff generation in the fastPHASE model. We specialize Algorithm 2 in the case of the fastPHASE model.[3] This HMM describes the distribution of genotypes as a patchwork of latent ancestral motifs. A quick preview of the results: after introducing the model, we first optimize the second step of Algorithm 2 to have complexity $\mathcal{O}(pK)$ for phased haplotypes, or $\mathcal{O}(pK^2)$ for unphased genotypes. Then, we optimize the first step of Algorithm 2 to have complexity $\mathcal{O}(pK)$ for phased haplotypes, or $\mathcal{O}(pK^2)$ for unphased genotypes. By combining these results, we decrease the complexity of Algorithm 2 to $\mathcal{O}(pK)$ for phased haplotypes, or $\mathcal{O}(pK^2)$ for unphased genotypes. This is an important contribution because it makes *KnockoffZoom* applicable to large datasets.

The fastPHASE model for phased haplotype sequences describes $\mathbf{X} = (X_1, \ldots, X_p)$, with $X_j \in \{0, 1\}$, as an imperfect mosaic of K ancestral motifs, $\mathbf{D}_i = (D_{i,1}, \ldots, D_{i,p}\}$, for $i \in \{1, \ldots, K\}$ and $D_{i,j} \in \{0, 1\}$. This can be formalized as an HMM with K hidden states, in the form of (5).[3] The transitions of the latent Markov chain are simple:

$$\mathbb{P}\left[Z_j = k \mid Z_{j-1} = l\right] = Q_j(k \mid l) = a_{j,k} + b_j \delta_{k,l}. \tag{7}$$

Above, $\delta_{k,l}$ indicates the Kronecker delta: $\delta_{k,l}$ is equal to 1 if $k = l$, and 0 otherwise. Conditional on $\mathbf{Z}$, each X_j is drawn independently from:

$$\mathbb{P}\left[X_j = 1 \mid \mathbf{Z}\right] = \mathbb{P}\left[X_j = 1 \mid Z_j\right] = \theta_{j,Z_j}.$$

The parameters $\theta = (\theta_{j,k})_{k \in [K], j \in [p]}$ describe the haplotype motifs in $\mathbf{D}$ and the mutation rates. We can write a and b consistent with the notation of fastPHASE[3] as:

$$a_{j,u} = \begin{cases} \alpha_{1,u}, & \text{if } j = 1, \\ \left(1 - e^{-r_j}\right) \alpha_{j,u}, & \text{if } j > 1, \end{cases} \qquad b_j = \begin{cases} 0, & \text{if } j = 1, \\ e^{-r_j}, & \text{if } j > 1. \end{cases}$$

The parameters $\alpha = (\alpha_{j,k})_{k \in [K], j \in [p]}$ describe the prevalence of each motif in the population. The likelihood of a transition in the Markov chain depends on the values of $r = (r_1, \ldots, r_p)$, which capture the genetic recombination rates along the genome. This phenomenological model of LD has inspired several successful applications for phasing and imputation.[4]

An unphased genotype sequence $\mathbf{X} = (X_1, \ldots, X_p)$, with $X_j \in \{0, 1, 2\}$, can be described as the element-wise sum of two independent and identically distributed haplotype sequences, H^a and H^b, that follow the model defined above. Consequently, $\mathbf{X}$ is also distributed as an HMM in the form of (5) with $K_{\text{eff}} = K(K+1)/2$ hidden states, where K is the number of haplotype motifs. This quadratic dependence on K follows from the fact that each latent Markov state of $\mathbf{X}$ corresponds to an unordered pair of states, $\{Z_j^a, Z_j^b\}$, corresponding to the unobserved haplotypes. The transition

probabilities for the effective Markov chain have the following structure:

$$\mathbb{P}\left[Z_j = \{k^a, k^b\} \mid Z_{j-1} = \{l^a, l^b\}\right] = \bar{Q}_j(\{k^a, k^b\} \mid \{l^a, l^b\})$$
$$= \begin{cases} Q_j(k^a \mid l^a)\, Q_j(k^b \mid l^b), & \text{if } k^a = k^b, \\ Q_j(k^a \mid l^a)\, Q_j(k^b \mid l^b) + Q_j(k^b \mid l^a)\, Q_j(k^a \mid l^b), & \text{otherwise.} \end{cases} \tag{8}$$

Above, $Q_j(k \mid l)$ is given by (7) while the conditional emission distributions are:

$$\mathbb{P}\left[X_j = x \mid \mathbf{Z} = \mathbf{z}\right] = \mathbb{P}\left[X_j = x \mid Z_j = \{k^a, k^b\}\right] = \begin{cases} (1-\theta_{j,k^a})(1-\theta_{j,k^b}), & \text{if } x = 0, \\ \theta_{j,k^a}(1-\theta_{j,k^b}) + (1-\theta_{j,k^a})\theta_{j,k^b}, & \text{if } x = 1, \\ \theta_{j,k^a}\theta_{j,k^b}, & \text{if } x = 2. \end{cases}$$

Generating group-knockoffs for genotypes using the general HMM algorithms would cost $\mathcal{O}(pK^6)$, while knockoffs for haplotypes would cost $\mathcal{O}(pK^3)$. This is prohibitive for large datasets, since the operation must be repeated separately for each subject. Before proceeding to simplify the algorithm analytically, we state the following useful lemma.

Lemma 1. *The Markov chain transition matrices for the unphased genotypes in the fastPHASE HMM can be written explicitly as:*

$$\bar{Q}_j(\{k^a, k^b\} \mid \{l^a, l^b\}) = a_{j,k^a} a_{j,k^b}(2 - \delta_{k^a,k^b}) + (b_j)^2 \delta_{\{l^a,l^b\},\{k^a,k^b\}} + b_j \frac{a_{j,k^a}\left(\delta_{k^b,l^a} + \delta_{k^b,l^b}\right) + a_{j,k^b}\left(\delta_{k^a,l^a} + \delta_{k^a,l^b}\right)}{1 + \delta_{k^a,k^b}}.$$

We leverage the phased haplotypes in the UK Biobank data[5] to accelerate the generation of the group-knockoffs for genotypes. Denote the haplotypes of one subject as $\mathbf{H}^a, \mathbf{H}^b \in \{0,1\}^p$, so that the genotypes are $\mathbf{X} = \mathbf{H}^a + \mathbf{H}^b \in \{0,1,2\}^p$. Only $\mathbf{X}$ is measures in a GWAS, whereas $\mathbf{H}^a$ and $\mathbf{H}^b$ are probabilistic reconstructions based on an HMM[4] similar to ours. Holding this thought, note that the following algorithm generates exact group-knockoffs for the genotypes: first, sample $\{\mathbf{H}^a, \mathbf{H}^b\}$ from their posterior distribution given $\mathbf{X}$; next, create group-knockoffs $\tilde{\mathbf{H}}^a$ for $\mathbf{H}^a$ and $\tilde{\mathbf{H}}^b$ for $\mathbf{H}^b$, independently; and lastly, set $\tilde{\mathbf{X}} = \tilde{\mathbf{H}}^a + \tilde{\mathbf{H}}^b$. The proof is equivalent to that of Proposition 2. The first step above corresponds to phasing (although sometimes phasing is carried out by reconstructing the most likely haplotypes $\mathbf{H}^a$ and $\mathbf{H}^b$, as opposed to posterior sampling). Given the reconstructed haplotypes, the second stage of the above algorithm only involves an HMM with K latent states, instead of $\mathcal{O}(K^2)$. The third stage of the algorithm is trivial.

We begin to specialize Algorithm 1 for the HMM of phased haplotypes, starting from its second step. Recall that the $\mathcal{N}$ functions in (4) are defined recursively as:

$$\mathcal{N}_g(k) = \sum_{z_1^g,\dots,z_{m_g}^g} \frac{Q_1^g(z_1^g \mid \tilde{z}_{m_{g-1}}^{g-1})^{\mathbb{1}[g\neq 1]}\, Q_1^g(z_1^g \mid z_{m_{g-1}}^{g-1})}{\mathcal{N}_{g-1}(z_1^g)} \times \left[\prod_{j=2}^{m_g} Q_j^g(z_j^g \mid z_{j-1}^g)\right] \times Q_1^{g+1}(k \mid z_{m_g}^g).$$

To simplify the notations, define, for each group g, $v^g \in \mathbb{R}^{m_g \times K}$ and $u^g \in \mathbb{R}^{m_g}$ as follows:

- The last row of v^g and the last element of u^g are:

$$v_{m_g,k}^g = a_{1,k}^{g+1}, \quad \forall k \in \{1,\dots,K\}, \qquad\qquad u_{m_g}^g = b_1^{g+1}.$$

- For $j \in \{1,\dots,m_g-1\}$, the j-th row of v^g and the j-th element of u^g are defined recursively:

$$v_{j,k}^g = v_{j+1,k}^g + u_{j+1}^g a_{j+1,k}^g, \quad \forall k \in \{1,\dots,K\}, \qquad\qquad u_j^g = u_{j+1}^g b_{j+1}^g.$$

Proposition 3. *For the special case of the fastPHASE model of unphased genotypes, the $\mathcal{N}$ function in Algorithm 1 for the g-th group* (4) *can be computed recursively as:*

$$\mathcal{N}_g(k) = u_1^g \frac{Q_1^g(k \mid \tilde{z}_{m_{g-1}}^{g-1})^{\mathbb{1}[g\neq 1]}\, Q_1^g(k \mid z_{m_{g-1}}^{g-1})}{\mathcal{N}_{g-1}(k)} + v_{1,k}^g \sum_{l=1}^{K} \frac{Q_1^g(l \mid \tilde{z}_{m_{g-1}}^{g-1})^{\mathbb{1}[g\neq 1]}\, Q_1^g(l \mid z_{m_{g-1}}^{g-1})}{\mathcal{N}_{g-1}(l)}. \qquad (9)$$

Above, it is understood that $\mathcal{N}_0(k) = 1$, $\forall k$.

Each $v_j^g \in \mathbb{R}^K$ can be computed in $\mathcal{O}(K)$ time, while the additional cost for u_j^g is $\mathcal{O}(1)$. Therefore, we compute v_1^g and u_1^g in $\mathcal{O}(m_g K)$ time and evaluate $\mathcal{N}_g(k)$ in $\mathcal{O}(m_g K)$ time (Proposition 3).

Given $\mathcal{N}_g$, we must sample the vector $\tilde{\mathbf{Z}}^g = (\tilde{Z}_1^g, \dots, \tilde{Z}_{m_g}^g)$ from:

$$\mathbb{P}\left[\tilde{\mathbf{Z}}^g = \tilde{z}^g \mid z^{-g}, \tilde{z}^{1:(g-1)}\right] \propto \frac{Q_1^g(\tilde{z}_1^g \mid \tilde{z}_{m_{g-1}}^{g-1})^{\mathbb{1}[g\neq 1]}\, Q_1^g(\tilde{z}_1^g \mid z_{m_{g-1}}^{g-1})}{\mathcal{N}_{g-1}(z_1^g)} \times \left[\prod_{j=2}^{m_g} Q_j^g(\tilde{z}_j^g \mid \tilde{z}_{j-1}^g)\right] \times Q_1^{g+1}(z_1^{g+1} \mid \tilde{z}_{m_g}^g).$$

This is a multivariate distribution from which we can sample efficiently, as stated next.

Proposition 4. *In the special case of Algorithm 1 for the fastPHASE model of phased haplotypes, the knockoff copy for the first element of the g-th group can be sampled from:*

$$\mathbb{P}\left[\tilde{Z}_1^g = k \mid z^{-g}, \tilde{z}^{1:(g-1)}\right] \propto \frac{Q_1^g(k \mid \tilde{z}_{m_{g-1}}^{g-1})^{\mathbb{1}[g\neq 1]}\, Q_1^g(k \mid z_{m_{g-1}}^{g-1})}{\mathcal{N}_{g-1}(k)} \left(v_{1,z_1^{g+1}}^g + u_1^g \mathbb{1}\left[k = z_1^{g+1}\right]\right).$$

For $j \in \{2,\dots,m_g\}$, the knockoff copy for the j-th element of the g-th group can be sampled from:

$$\mathbb{P}\left[\tilde{Z}_j^g = k \mid z^{-g}, \tilde{z}^{1:(g-1)}, \tilde{z}_{1:(j-1)}^g\right] \propto Q_j^g(k \mid \tilde{z}_{j-1}^g) \left(v_{j,z_1^{g+1}}^g + u_j^g \mathbb{1}\left[k = z_1^{g+1}\right]\right).$$

Each coordinate is sampled at an additional cost $\mathcal{O}(K)$, reusing v_j^g, u_j^g from the computation of the $\mathcal{N}$ functions. The cost for the sequence is $\mathcal{O}(\sum_{g=1}^G m_g K) = \mathcal{O}(pK)$, regardless of the grouping.

We perform analogous calculations in the case of the HMM for unphased genotypes. Here the notation is more involved. The functions in (4) are defined recursively as:

$$\mathcal{N}_g(\{k^a,k^b\}) = \sum_{\{l_1^a,l_1^b\},\ldots,\{l_{m_g}^a,l_{m_g}^b\}} \frac{\bar{Q}_1^g(\{l_1^a,l_1^b\} \mid z_{m_{g-1}}^{g-1})\,\bar{Q}_1^g(\{l_1^a,l_1^b\} \mid \tilde{z}_{m_{g-1}}^{g-1})^{\mathbb{1}[g\neq 1]}}{\mathcal{N}_{g-1}(\{l_1^a,l_1^b\})} \times \left[\prod_{j=2}^{m_g} \bar{Q}_j^g(\{l_j^a,l_j^b\} \mid \{l_{j-1}^a,l_{j-1}^b\})\right] \times \bar{Q}_1^{g+1}(\{k^a,k^b\} \mid \{l_{m_g}^a,l_{m_g}^b\}).$$

We start by defining some new variables. For $k, k^a, k^b \in \{1,\ldots,K\}$, let:

$$u_{m_g}^g = (b_1^{g+1})^2, \qquad v_{m_g,\{k^a,k^b\}}^g = a_{1,k^a}^{g+1} a_{1,k^b}^{g+1}, \qquad w_{m_g,k}^g = b_1^{g+1} a_{1,k}^{g+1}.$$

For $j \in \{1,\ldots,m_g-1\}$ and $k^a, k^b \in \{1,\ldots,K\}$, we define recursively:

$$\begin{aligned} u_j^g &= u_{j+1}^g (b_{j+1}^g)^2, \\ v_{j,\{k^a,k^b\}}^g &= v_{j+1,\{k^a,k^b\}}^g + w_{j+1,k^a}^g a_{j+1,k^b}^g + w_{j+1,k^b}^g a_{j+1,k^a}^g + u_{j+1}^g a_{j+1,k^a}^g a_{j+1,k^b}^g, \\ w_{j,k}^g &= w_{j+1,k}^g b_{j+1}^g + u_{j+1}^g a_{j+1,k}^g b_{j+1}^g. \end{aligned}$$

Moreover, we also define:

$$C^g(\{k^a,k^b\}) = \frac{\bar{Q}_1^g(\{k^a,k^b\} \mid \tilde{z}_{m_{g-1}}^{g-1})^{\mathbb{1}[g\neq 1]}\,\bar{Q}_1^g(\{k^a,k^b\} \mid z_{m_{g-1}}^{g-1})}{\mathcal{N}_{g-1}(\{k^a,k^b\})},$$

and

$$D^g(k^a) = C^g(k^a,k^a) + \sum_{k^b=1}^K C^g(\{k^a,k^b\}).$$

The following result shows that the cost of computing the g-th $\mathcal{N}$ function is $\mathcal{O}(m_g K^2)$.

Proposition 5. *For the special case of the fastPHASE model of unphased genotypes, the $\mathcal{N}$ function in Algorithm 1 for the g-th group is given by:*

$$\mathcal{N}_g(\{k^a,k^b\}) = \frac{w_{1,k^a}^g D^g(k^b) + w_{1,k^b}^g D^g(k^a)}{1+\delta_{k^a,k^b}} + u_1^g\, C^g(\{k^a,k^b\}) + (2-\delta_{k^a,k^b}) v_{1,\{k^a,k^b\}}^g \sum_{\{l^a,l^b\}} C^g(\{l^a,l^b\}).$$

The sampling part of Algorithm 1 is similar to what we saw earlier.

Proposition 6. *For the special case of the fastPHASE model of unphased genotypes, the knockoff copy for the first element of the g-th group can be sampled in Algorithm 1 from:*

$$\mathbb{P}\left[\tilde{Z}_1^g = \{k^a, k^b\} \mid z^{-g}, \tilde{z}^{1:(g-1)}\right] \propto \frac{\bar{Q}_1^g(\{k^a, k^b\} \mid \tilde{z}_{m_{g-1}}^{g-1})^{\mathbb{1}[g\neq 1]}\, \bar{Q}_1^g(\{k^a, k^b\} \mid z_{m_{g-1}}^{g-1})}{\mathcal{N}_{g-1}(\{k^a, k^b\})} V_1^g(z_1^{g+1} \mid \{k^a, k^b\}).$$

The knockoff for the j-th element of the g-th group, for $j \in \{2, \ldots, m_g\}$, can be sampled from:

$$\mathbb{P}\left[\tilde{Z}_j^g = \{k^a, k^b\} \mid z^{-g}, \tilde{z}^{1:(g-1)}\right] \propto \bar{Q}_j^g(\{k^a, k^b\} \mid \tilde{z}_{j-1}^g)\, V_j^g(z_1^{g+1} \mid \{k^a, k^b\}).$$

Above, the variables V_j^g are defined as:

$$\begin{aligned} V_j^g(\{l^a, l^b\} \mid \{k^a, k^b\}) = {} & v_{j,\{l^a,l^b\}}^g(2 - \delta_{l^a,l^b}) + u_j^g \delta_{\{l^a,l^b\},\{k^a,k^b\}} \\ & + \frac{w_{j,l^a}^g \left(\delta_{l^b,k^a} + \delta_{l^b,k^b}\right) + w_{j,l^b}^g \left(\delta_{l^a,k^a} + \delta_{l^a,k^b}\right)}{1 + \delta_{l^a,l^b}}. \end{aligned}$$

Sampling group-knockoffs costs $\mathcal{O}(\sum_{g=1}^G m_g K^2) = \mathcal{O}(pK^2)$ per individual, with any grouping.

The first step in Algorithm 2 consists of sampling $\mathbf{Z}$ from the posterior distribution of the latent Markov chain in (5) given $\mathbf{X}$. In general, this requires a variation of Viterbi's algorithm,[2] as recalled in Algorithms 3 and 4. Here, K indicates the number of states in the Markov chain.

Algorithm 3 Forward-backward sampling (forward pass)

Initialize $F_0 = 1, \quad Q_1(k \mid l) = Q_1(k)$, for all k, l
for $j = 1$ to p **do**
  for $k = 1$ to K **do**
    Compute $F_j(k) = f_j(x_j \mid k) \sum_{l=1}^K Q_j(k \mid l)\, F_{j-1}(l)$.

Algorithm 4 Forward-backward sampling (backward pass)

Initialize $j = p, \quad Q_{p+1}(k \mid l) = 1$ for all k, l
for $j = p$ to 1 (backward) **do**
  Sample Z_j from $\mathbb{P}[Z_j = k] = \frac{Q_{j+1}(Z_{j+1}|k)\, F_j(k)}{\sum_{l=1}^K Q_{j+1}(Z_{j+1}|l)\, F_j(l)}$.

The forward probabilities in Algorithm 3 are defined recursively, for $j \in \{2, \ldots, p\}$, as:

$$\begin{aligned} F_1(k) &= f_1(x_1 \mid k)\, Q_1(k), \\ F_{j+1}(k) &= f_{j+1}(x_{j+1} \mid k) \sum_{l=1}^K Q_{j+1}(k \mid l)\, F_j(l). \end{aligned}$$

The cost of computing all forward probabilities is generally $\mathcal{O}(pK^2)$ but it can be reduced to $\mathcal{O}(pK)$ using the symmetries in (7). Earlier instances of the same idea can be found in the statistical genetics literature,[6] although we prefer to present the full results here for completeness, especially since they are easy to present using the notation developed so far.

Proposition 7. *In the special case of the fastPHASE model of phased haplotypes, the forward probabilities in Algorithm 3 can be computed recursively as follows:*

$$\begin{aligned} F_1(k) &= f_1(x_1 \mid k)\, a_{1,k}, \\ F_{j+1}(k) &= f_{j+1}(x_{j+1} \mid k) \left[a_{j+1,k} \sum_{l=1}^{K} F_j(l) + b_{j+1} F_j(k) \right]. \end{aligned}$$

Above, the sum only needs be computed once because it does not depend on the value k. Consequently, we can implement the first part of Algorithm 3 at cost $\mathcal{O}(pK)$.

We can also implement Algorithm 3 efficiently for unphased genotypes. For $j \in \{2, \dots, p\}$, the forward probabilities are defined as:

$$\begin{aligned} F_1(\{k^a, k^b\}) &= f_1(x_1 \mid \{k^a, k^b\})\, \bar{Q}_1(\{k^a, k^b\}), \\ F_{j+1}(\{k^a, k^b\}) &= f_{j+1}(x_{j+1} \mid \{k^a, k^b\}) \sum_{\{l^a, l^b\}} \bar{Q}_{j+1}(\{k^a, k^b\} \mid \{l^a, l^b\})\, F_j(\{l^a, l^b\}). \end{aligned}$$

This computation generally costs $\mathcal{O}(pK^4)$ because the number of discrete states in the latent Markov chain is quadratic in the number of haplotype motifs.

Proposition 8. *In the special case of the fastPHASE model of unphased genotypes, the forward probabilities in Algorithm 3 are given by the following recursive formula:*

$$\begin{aligned} \frac{F_1(\{k^a, k^b\})}{f_1(x_1 \mid \{k^a, k^b\})} &= a_{1,k^a} a_{1,k^b} (2 - \delta_{k^a,k^b}), \\ \frac{F_{j+1}(\{k^a, k^b\})}{f_{j+1}(x_{j+1} \mid \{k^a, k^b\})} &= (b_{j+1})^2 F_j(\{k^a, k^b\}) + (2 - \delta_{k^a,k^b}) \left[a_{j+1,k^a} a_{j+1,k^b} \sum_{\{l^a, l^b\}} F_j(\{l^a, l^b\}) \right] \\ &\quad + b_{j+1} \frac{a_{j+1,k^a}}{1 + \delta_{k^a,k^b}} \left[F_j(\{k^b, k^b\}) + \sum_{l=1}^{K} F_j(\{l, k^b\}) \right] \\ &\quad + b_{j+1} \frac{a_{j+1,k^b}}{1 + \delta_{k^a,k^b}} \left[F_j(\{k^a, k^a\}) + \sum_{l=1}^{K} F_j(\{l, k^a\}) \right]. \end{aligned}$$

This gives us a $\mathcal{O}(pK^2)$ algorithm because the above sums can be efficiently pre-computed.

Mathematical proofs

Proof of Proposition 1. Our algorithm implements the SCIP recipe,[15] which provably generates valid knockoffs, for the vector-valued random variables $\mathbf{Z}^1, \ldots, \mathbf{Z}^L$. Therefore, it suffices to show that (3) gives a correct expression for $p(\mathbf{Z}^g \mid \mathbf{Z}^{-g}, \tilde{\mathbf{Z}}^{1:(g-1)})$. To this end, we proceed by induction. Assuming that (3) holds $\forall g \in \{1, \ldots, g'-1\}$, for some $g' > 1$, it follows that:

$$
\begin{aligned}
&p(\mathbf{Z}^{g'} | \mathbf{Z}^{-g'}, \tilde{\mathbf{Z}}^{1:(g'-1)}) \\
&\qquad \propto p(\mathbf{Z}^{g'}, \mathbf{Z}^{-g'}) p(\tilde{\mathbf{Z}}^{1:(g'-1)} \mid \mathbf{Z}^{g'}, \mathbf{Z}^{-g'}) \\
&\qquad \propto Q_1^{g'}(Z_1^{g'} \mid Z_{m_{g'-1}}^{g'-1}) \left[\prod_{j=2}^{m_{g'}} Q_j^{g'}(Z_j^{g'} \mid Z_{j-1}^{g'}) \right] Q_1^{g'+1}(Z_1^{g'+1} \mid Z_{m'_g}^{g'}) p(\mathbf{Z}^{g'-1} \mid Z^{g'}, Z^{-g'}) \\
&\qquad \propto Q_1^{g'}(Z_1^{g'} \mid Z_{m_{g'-1}}^{g'-1}) \left[\prod_{j=2}^{m_{g'}} Q_j^{g'}(Z_j^{g'} \mid Z_{j-1}^{g'}) \right] Q_1^{g'+1}(Z_1^{g'+1} \mid Z_{m'_g}^{g'}) \frac{Q_1^{g'}(Z_1^{g'} \mid \tilde{Z}_{m_{g'-1}}^{g'-1})}{\mathcal{N}_{g'-1}(Z_1^{g'})}.
\end{aligned}
$$

Above, the proportionality holds across values of the vector $\mathbf{Z}^{g'}$. In view of the normalization in (4), we conclude that (3) gives a correct expression for $p(\mathbf{Z}^g \mid \mathbf{Z}^{-g}, \tilde{\mathbf{Z}}^{1:(g-1)})$ for all $g \in \{1, \ldots, g'\}$. Since the base case with $g = 1$ clearly holds, the proof is complete. □

Proof of Lemma 1. This follows immediately by replacing (7) into (8) and simplifying. □

Proof of Proposition 2. It suffices to prove (6), since marginalizing over $(\mathbf{Z}, \tilde{\mathbf{Z}})$ implies that $(\mathbf{X}, \tilde{\mathbf{X}})_{\text{swap}(G;\mathcal{G})}$ has the same distribution as $(\mathbf{X}, \tilde{\mathbf{X}})$.[2] Conditioning on the latent variables yields:

$$
\begin{aligned}
&\mathbb{P}\Big[(\mathbf{X},\tilde{\mathbf{X}}) = (\mathbf{x}, \tilde{\mathbf{x}})_{\text{swap}(G;\mathcal{G})} \mid (\mathbf{Z}, \tilde{\mathbf{Z}}) = (\mathbf{z}, \tilde{\mathbf{z}})_{\text{swap}(G;\mathcal{G})}\Big] \\
&\qquad = \mathbb{P}\left[(\mathbf{X}, \tilde{\mathbf{X}}) = (\mathbf{x}, \tilde{\mathbf{x}})_{\text{swap}(G;\mathcal{G})} \middle| (\mathbf{Z}, \tilde{\mathbf{Z}}) = (\mathbf{z}, \tilde{\mathbf{z}})_{\text{swap}(G;\mathcal{G})} \right] \mathbb{P}\left[(\mathbf{Z}, \tilde{\mathbf{Z}}) = (\mathbf{z}, \tilde{\mathbf{z}})_{\text{swap}(G;\mathcal{G})} \right] \\
&\qquad = \mathbb{P}\left[(\mathbf{X}, \tilde{\mathbf{X}}) = (\mathbf{x}, \tilde{\mathbf{x}}) \middle| (\mathbf{Z}, \tilde{\mathbf{Z}}) = (\mathbf{z}, \tilde{\mathbf{z}}) \right] \mathbb{P}\left[(\mathbf{Z}, \tilde{\mathbf{Z}}) = (\mathbf{z}, \tilde{\mathbf{z}})_{\text{swap}(G;\mathcal{G})} \right] \\
&\qquad = \mathbb{P}\left[(\mathbf{X}, \tilde{\mathbf{X}}) = (\mathbf{x}, \tilde{x}) \middle| (\mathbf{Z}, \tilde{\mathbf{Z}}) = (\mathbf{z}, \tilde{\mathbf{z}}) \right] \mathbb{P}\left[(\mathbf{Z}, \tilde{\mathbf{Z}}) = (\mathbf{z}, \tilde{\mathbf{z}}) \right].
\end{aligned}
$$

Above, the first equality follows from the first line of Algorithm 2, the second from the conditional independence of the emission distributions in an HMM, and the third from Proposition 1). □

Proof of Proposition 3. The $\mathcal{N}$ function for the g-th group can be written as:

$$
\begin{aligned}
\mathcal{N}_g(k) = &\sum_{z_1^g, \ldots, z_{m_g-1}^g} \frac{Q_1^g(z_1^g \mid \tilde{z}_{m_{g-1}}^{g-1})^{\mathbb{1}[g \neq 1]} \, Q_1^g(z_1^g \mid z_{m_{g-1}}^{g-1})}{\mathcal{N}_{g-1}(z_1^g)} \times \left[\prod_{j=2}^{m_g-1} Q_j^g(z_j^g \mid z_{j-1}^g) \right] \\
&\times \sum_{z_{m_g}^g} Q_{m_g}^g(z_{m_g}^g \mid z_{m_g-1}^g) \, Q_1^{g+1}(k \mid z_{m_g}^g).
\end{aligned}
$$

To simplify the notation, we define $V^g_{m_g}(k \mid l) = Q^{g+1}_1(k \mid l)$, and, recursively for $j \in \{1, \ldots, m_g - 1\}$,

$$V^g_j(k \mid l) = \sum_{l'=1}^{K} Q^g_{j+1}(l' \mid l)\, V^g_{j+1}(k \mid l').$$

Then, we see that the $\mathcal{N}$ function can be reduced to:

$$\mathcal{N}_g(k) = \sum_{l=1}^{K} \frac{Q^g_1(l \mid \tilde{z}^{g-1}_{m_{g-1}})^{\mathbb{1}[g \neq 1]}\, Q^g_1(l \mid z^{g-1}_{m_{g-1}})}{\mathcal{N}_{g-1}(l)} V^g_1(k \mid l).$$

It remains to be shown how to compute $V^g_1(k \mid l)$. We guess that $V^g_j(k \mid l)$ can be written as:

$$V^g_j(k \mid l) = v^g_{j,k} + u^g_j \mathbb{1}\left[k = l\right],$$

since $Q^{g+1}_1(k \mid l) = a^{g+1}_{1,k} + b^{g+1}_1 \mathbb{1}\left[k = l\right]$. This ansatz is clearly correct if $j = m_g$, by definition of $V^g_{m_g}(k \mid l)$, in which case: $v^g_{m_g,k} = a^{g+1}_{1,k}$ and $u^g_{m_g} = b^{g+1}_1$. When $j \in \{1, \ldots, m_g - 1\}$, backward induction shows that

$$\begin{aligned}
V^g_j(k' \mid k) &= \sum_{l=1}^{K} Q^g_{j+1}(l \mid k)\, V^g_{j+1}(k' \mid l) \\
&= \sum_{l=1}^{K} Q^g_{j+1}(l \mid k) \left(v^g_{j+1,k'} + u^g_{j+1} \mathbb{1}\left[k' = l\right]\right) \\
&= v^g_{j+1,k'} \sum_{l=1}^{K} Q^g_{j+1}(l \mid k) + u^g_{j+1} Q^g_{j+1}(k' \mid k) \\
&= v^g_{j+1,k'} \sum_{l=1}^{K} \left(a^g_{j+1,l} + b^g_{j+1} \mathbb{1}\left[l = k\right]\right) + u^g_{j+1} \left(a^g_{j+1,k'} + b^g_{j+1} \mathbb{1}\left[k' = k\right]\right) \\
&= v^g_{j+1,k'} \left(\sum_{l=1}^{K} a^g_{j+1,l} + b^g_{j+1}\right) + u^g_{j+1} a^g_{j+1,k'} + u^g_{j+1} b^g_{j+1} \mathbb{1}\left[k' = k\right]. \\
&= v^g_{j+1,k'} + u^g_{j+1} a^g_{j+1,k'} + u^g_{j+1} b^g_{j+1} \mathbb{1}\left[k' = k\right].
\end{aligned}$$

Therefore, our guess is correct for j, as long as it is for $j + 1$, since

$$v^g_{j,k'} = v^g_{j+1,k'} + u^g_{j+1} a^g_{j+1,k'}, \qquad u^g_j = u^g_{j+1} b^g_{j+1}.$$

□

Proof of Proposition 4. We start from

$$\mathbb{P}\left[\tilde{\mathbf{Z}}^g = \tilde{z}^g \mid z^{-g}, \tilde{z}^{1:(g-1)}\right] \propto \frac{Q^g_1(\tilde{z}^g_1 \mid \tilde{z}^{g-1}_{m_{g-1}})^{\mathbb{1}[g \neq 1]}\, Q^g_1(\tilde{z}^g_1 \mid z^{g-1}_{m_{g-1}})}{\mathcal{N}_{g-1}(z^g_1)} \left[\prod_{j=2}^{m_g} Q^g_j(\tilde{z}^g_j \mid \tilde{z}^g_{j-1})\right]$$
$$\times\, Q^{g+1}_1(z^{g+1}_1 \mid \tilde{z}^g_{m_g}),$$

and marginalize over the remaining $m_g - 1$ components, finding:

$$\mathbb{P}\left[\tilde{Z}_1^g = k \mid z^{-g}, \tilde{z}^{1:(g-1)}\right] \propto \frac{Q_1^g(k \mid \tilde{z}_{m_{g-1}}^{g-1})^{\mathbb{1}[g \neq 1]}\, Q_1^g(k \mid z_{m_{g-1}}^{g-1})}{\mathcal{N}_{g-1}(k)} V_1^g(z_1^{g+1} \mid k).$$

Above, V_j^g is as defined in the proof of Proposition 3. Given $\tilde{Z}_1^g$, we can sample each $\tilde{Z}_j^g$ sequentially, from $j = 2$ to $j = m_g$. It is easy to verify that, at the j-th step, we need to sample $\tilde{Z}_j^g$ from:

$$\mathbb{P}\left[\tilde{Z}_j^g = k \mid z^{-g}, \tilde{z}^{1:(g-1)}, \tilde{z}_{1:(j-1)}^g\right] \propto Q_j^g(k \mid \tilde{z}_{j-1}^g)\, V_j^g(z_1^{g+1} \mid k).$$

□

Proof of Proposition 5. Starting from

$$\mathcal{N}_g(\{k^a, k^b\}) = \sum_{\{l_1^a, l_1^b\}, \dots, \{l_{m_g}^a, l_{m_g}^b\}} \frac{\bar{Q}_1^g(\{l_1^a, l_1^b\} \mid z_{m_{g-1}}^{g-1})^{\mathbb{1}[g \neq 1]}\, \bar{Q}_1^g(\{l_1^a, l_1^b\} \mid \tilde{z}_{m_{g-1}}^{g-1})}{\mathcal{N}_{g-1}(\{l_1^a, l_1^b\})} \times \left[\prod_{j=2}^{m_g} \bar{Q}_j^g(\{l_j^a, l_j^b\} \mid \{l_{j-1}^a, l_{j-1}^b\})\right] \times \bar{Q}_1^{g+1}(\{k^a, k^b\} \mid \{l_{m_g}^a, l_{m_g}^b\}),$$

we proceed as in the proof of Proposition 3, defining:

$$V_{m_g}^g(\{k^a, k^b\} \mid \{l^a, l^b\}) = \bar{Q}_1^{g+1}(\{k^a, k^b\} \mid \{l^a, l^b\}).$$

Similarly, for $j = 1, \dots, m_g - 1$, we recursively define:

$$V_j^g(\{k^a, k^b\} \mid \{l^a, l^b\}) = \sum_{\{h^a, h^b\}} \bar{Q}_{j+1}^g(\{h^a, h^b\} \mid \{l^a, l^b\})\, V_{j+1}^g(\{k^a, k^b\} \mid \{h^a, h^b\}).$$

Using Lemma 1, we can write:

$$V_{m_g}^g(\{k^a, k^b\} \mid \{l^a, l^b\}) = a_{1,k^a}^{g+1} a_{1,k^b}^{g+1}(2 - \delta_{k^a,k^b}) + (b_1^{g+1})^2 \delta_{\{k^a,k^b\},\{l^a,l^b\}} + b_1^{g+1} \frac{a_{1,k^a}^{g+1}\left(\delta_{k^b,l^a} + \delta_{k^b,l^b}\right) + a_{1,k^b}^{g+1}\left(\delta_{k^a,l^a} + \delta_{k^a,l^b}\right)}{1 + \delta_{k^a,k^b}}.$$

It is now time for an ansatz. For $j = 1, \dots, m_g - 1$, we guess that:

$$V_j^g(\{k^a, k^b\} \mid \{l^a, l^b\}) = v_{j,\{k^a,k^b\}}^g(2 - \delta_{k^a,k^b}) + u_j^g \delta_{\{k^a,k^b\},\{l^a,l^b\}} + \frac{w_{j,k^a}^g\left(\delta_{k^b,l^a} + \delta_{k^b,l^b}\right) + w_{j,k^b}^g\left(\delta_{k^a,l^a} + \delta_{k^a,l^b}\right)}{1 + \delta_{k^a,k^b}}.$$

Assuming that our guess is correct for $j + 1$, with some $j \in \{1, \dots, m_g - 1\}$, we can write:

$$V_j^g(\{k^a, k^b\} \mid \{l^a, l^b\}) = \sum_{\{h^a, h^b\}} \bar{Q}_{j+1}^g(\{h^a, h^b\} \mid \{l^a, l^b\})\, V_{j+1}^g(\{k^a, k^b\} \mid \{h^a, h^b\})$$

$$
\begin{aligned}
&= v^g_{j+1,\{k^a,k^b\}}(2-\delta_{k^a,k^b}) \sum_{\{h^a,h^b\}} \bar{Q}^g_{j+1}(\{h^a,h^b\} \mid \{l^a,l^b\}) \\
&\quad + \frac{w^g_{j+1,k^a}}{1+\delta_{k^a,k^b}} \sum_{\{h^a,h^b\}} \left(\delta_{k^b,h^a}+\delta_{k^b,h^b}\right) \bar{Q}^g_{j+1}(\{h^a,h^b\} \mid \{l^a,l^b\}) \\
&\quad + \frac{w^g_{j+1,k^b}}{1+\delta_{k^a,k^b}} \sum_{\{h^a,h^b\}} \left(\delta_{k^a,h^a}+\delta_{k^a,h^b}\right) \bar{Q}^g_{j+1}(\{h^a,h^b\} \mid \{l^a,l^b\}) \\
&\quad + u^g_{j+1}\,\bar{Q}^g_{j+1}(\{k^a,k^b\} \mid \{l^a,l^b\}) \\
&= v^g_{j+1,\{k^a,k^b\}}(2-\delta_{k^a,k^b}) + u^g_{j+1}\,\bar{Q}^g_{j+1}(\{k^a,k^b\} \mid \{l^a,l^b\}) \\
&\quad + \frac{1}{1+\delta_{k^a,k^b}} \left[w^g_{j+1,k^a}\Gamma(k^b \mid \{l^a,l^b\}) + w^g_{j+1,k^b}\Gamma(k^a \mid \{l^a,l^b\})\right].
\end{aligned}
$$

Above, we have defined:

$$
\Gamma(k \mid \{l^a,l^b\}) = \sum_{\{h^a,h^b\}} \left(\delta_{k,h^a}+\delta_{k,h^b}\right) \bar{Q}^g_{j+1}(\{h^a,h^b\} \mid \{l^a,l^b\}).
$$

This can be further simplified:

$$
\begin{aligned}
\Gamma(k \mid \{l^a,l^b\}) &= \sum_{\{h^a,h^b\}} \left(\delta_{k,h^a}+\delta_{k,h^b}\right) \bar{Q}^g_{j+1}(\{h^a,h^b\} \mid \{l^a,l^b\}) \\
&= 2\sum_h \delta_{k,h}\bar{Q}^g_{j+1}(\{h,h\} \mid \{l^a,l^b\}) + \sum_{\{h^a,h^b\},h^a\neq h^b} \left(\delta_{k,h^a}+\delta_{k,h^b}\right) \bar{Q}^g_{j+1}(\{h^a,h^b\} \mid \{l^a,l^b\}) \\
&= 2\bar{Q}^g_{j+1}(\{k,k\} \mid \{l^a,l^b\}) + \sum_{\{h^a,h^b\},h^a\neq h^b} \left(\delta_{k,h^a}+\delta_{k,h^b}\right) \bar{Q}^g_{j+1}(\{h^a,h^b\} \mid \{l^a,l^b\}) \\
&= 2\bar{Q}^g_{j+1}(\{k,k\} \mid \{l^a,l^b\}) + \frac{1}{2} \sum_{h^a,h^b,h^a\neq h^b} \left(\delta_{k,h^a}+\delta_{k,h^b}\right) \bar{Q}^g_{j+1}(\{h^a,h^b\} \mid \{l^a,l^b\}) \\
&= 2\bar{Q}^g_{j+1}(\{k,k\} \mid \{l^a,l^b\}) + \frac{1}{2} \sum_{h^a,h^b,h^a\neq h^b} \left(\delta_{k,h^a}+\delta_{k,h^b}\right) Q^g_{j+1}(h^a \mid l^a) Q^g_{j+1}(h^b \mid l^b) \\
&\quad + \frac{1}{2} \sum_{h^a,h^b,h^a\neq h^b} \left(\delta_{k,h^a}+\delta_{k,h^b}\right) Q^g_{j+1}(h^a \mid l^b) Q^g_{j+1}(h^b \mid l^a) \\
&= 2\bar{Q}^g_{j+1}(\{k,k\} \mid \{l^a,l^b\}) + \frac{1}{2} Q^g_{j+1}(k \mid l^a) \sum_{h^b\neq k} Q^g_{j+1}(h^b \mid l^b) \\
&\quad + \frac{1}{2} Q^g_{j+1}(k \mid l^b) \sum_{h^a\neq k} Q^g_{j+1}(h^a \mid l^a) + \frac{1}{2} Q^g_{j+1}(k \mid l^b) \sum_{h^b\neq k} Q^g_{j+1}(h^b \mid l^a) \\
&\quad + \frac{1}{2} Q^g_{j+1}(k \mid l^a) \sum_{h^a\neq k} Q^g_{j+1}(h^a \mid l^b) \\
&= 2\bar{Q}^g_{j+1}(\{k,k\} \mid \{l^a,l^b\}) \\
&\quad + \frac{1}{2} Q^g_{j+1}(k \mid l^a) \left[1 - Q^g_{j+1}(k \mid l^b)\right] + \frac{1}{2} Q^g_{j+1}(k \mid l^b) \left[1 - Q^g_{j+1}(k \mid l^a)\right] \\
&\quad + \frac{1}{2} Q^g_{j+1}(k \mid l^b) \left[1 - Q^g_{j+1}(k \mid l^a)\right] + \frac{1}{2} Q^g_{j+1}(k \mid l^a) \left[1 - Q^g_{j+1}(k \mid l^b)\right]
\end{aligned}
$$

$$
\begin{aligned}
&= 2Q^g_{j+1}(k \mid l^a)Q^g_{j+1}(k \mid l^b) + Q^g_{j+1}(k \mid l^a)\left[1 - Q^g_{j+1}(k \mid l^b)\right] \\
&\quad + Q^g_{j+1}(k \mid l^b)\left[1 - Q^g_{j+1}(k \mid l^a)\right] \\
&= Q^g_{j+1}(k \mid l^a) + Q^g_{j+1}(k \mid l^b) \\
&= 2a^g_{j+1,k} + b^g_{j+1}\left(\delta_{k,l^a} + \delta_{k,l^b}\right).
\end{aligned}
$$

Therefore, we can rewrite V^g_j, as follows:

$$
\begin{aligned}
&V^g_j(\{k^a, k^b\} \mid \{l^a, l^b\}) \\
&\quad = v^g_{j+1,\{k^a,k^b\}}(2 - \delta_{k^a,k^b}) + u^g_{j+1}\,\bar{Q}^g_{j+1}(\{k^a, k^b\} \mid \{l^a, l^b\}) \\
&\qquad + \frac{w^g_{j+1,k^a}\Gamma(k^b \mid \{l^a, l^b\}) + w^g_{j+1,k^b}\Gamma(k^a \mid \{l^a, l^b\})}{1 + \delta_{k^a,k^b}} \\
&\quad = v^g_{j+1,\{k^a,k^b\}}(2 - \delta_{k^a,k^b}) \\
&\qquad + \frac{w^g_{j+1,k^a}\left[2a^g_{j+1,k^b} + b^g_{j+1}\left(\delta_{k^b,l^a} + \delta_{k^b,l^b}\right)\right] + w^g_{j+1,k^b}\left[2a^g_{j+1,k^a} + b^g_{j+1}\left(\delta_{k^a,l^a} + \delta_{k^a,l^b}\right)\right]}{1 + \delta_{k^a,k^b}} \\
&\qquad + u^g_{j+1}\Bigg[a_{j+1,k^a}a_{j+1,k^b}(2 - \delta_{k^a,k^b}) + (b^g_{j+1})^2\delta_{\{l^a,l^b\},\{k^a,k^b\}} \\
&\qquad\qquad + b^g_{j+1}\frac{a^g_{j+1,k^a}\left(\delta_{k^b,l^a} + \delta_{k^b,l^b}\right) + a^g_{j+1,k^b}\left(\delta_{k^a,l^a} + \delta_{k^a,l^b}\right)}{1 + \delta_{k^a,k^b}}\Bigg].
\end{aligned}
$$

Now, we only need to collect these terms to obtain the recursion rules:

$$
\begin{aligned}
u^g_j &= u^g_{j+1}(b^g_{j+1})^2, \\
v^g_{j,\{k^a,k^b\}} &= v^g_{j+1,\{k^a,k^b\}} + u^g_{j+1}a^g_{j+1,k^a}a^g_{j+1,k^b} + w^g_{j+1,k^a}a^g_{j+1,k^b} + w^g_{j+1,k^b}a^g_{j+1,k^a}, \\
w^g_{j,k} &= w^g_{j+1,k}b^g_{j+1} + u^g_{j+1}a^g_{j+1,k}b^g_{j+1}.
\end{aligned}
$$

Finally, the $\mathcal{N}$ function for the g-th group is given by:

$$
\begin{aligned}
\mathcal{N}_g(\{k^a, k^b\}) &= \sum_{\{l^a,l^b\}} \frac{\bar{Q}^g_1(\{l^a, l^b\} \mid z^{g-1}_{m_{g-1}})\,\bar{Q}^g_1(\{l^a, l^b\} \mid \tilde{z}^{g-1}_{m_{g-1}})^{\mathbb{1}[g\neq 1]}}{\mathcal{N}_{g-1}(\{l^a, l^b\})} V^g_1(\{k^a, k^b\} \mid \{l^a, l^b\}) \\
&= v^g_{1,\{k^a,k^b\}}(2 - \delta_{k^a,k^b}) \sum_{\{l^a,l^b\}} \frac{\bar{Q}^g_1(\{l^a, l^b\} \mid z^{g-1}_{m_{g-1}})\,\bar{Q}^g_1(\{l^a, l^b\} \mid \tilde{z}^{g-1}_{m_{g-1}})^{\mathbb{1}[g\neq 1]}}{\mathcal{N}_{g-1}(\{l^a, l^b\})} \\
&\quad + \frac{w^g_{1,k^a}}{1 + \delta_{k^a,k^b}} \sum_{\{l^a,l^b\}} \frac{\bar{Q}^g_1(\{l^a, l^b\} \mid z^{g-1}_{m_{g-1}})\,\bar{Q}^g_1(\{l^a, l^b\} \mid \tilde{z}^{g-1}_{m_{g-1}})^{\mathbb{1}[g\neq 1]}}{\mathcal{N}_{g-1}(\{l^a, l^b\})}\left(\delta_{k^b,l^a} + \delta_{k^b,l^b}\right) \\
&\quad + \frac{w^g_{1,k^b}}{1 + \delta_{k^a,k^b}} \sum_{\{l^a,l^b\}} \frac{\bar{Q}^g_1(\{l^a, l^b\} \mid z^{g-1}_{m_{g-1}})\,\bar{Q}^g_1(\{l^a, l^b\} \mid \tilde{z}^{g-1}_{m_{g-1}})^{\mathbb{1}[g\neq 1]}}{\mathcal{N}_{g-1}(\{l^a, l^b\})}\left(\delta_{k^a,l^a} + \delta_{k^a,l^b}\right) \\
&\quad + u^g_1\frac{\bar{Q}^g_1(\{k^a, k^b\} \mid z^{g-1}_{m_{g-1}})\,\bar{Q}^g_1(\{k^a, k^b\} \mid \tilde{z}^{g-1}_{m_{g-1}})^{\mathbb{1}[g\neq 1]}}{\mathcal{N}_{g-1}(\{k^a, k^b\})}.
\end{aligned}
$$

Let us now define

$$C^g(\{l^a, l^b\}) = \frac{\bar{Q}_1^g(\{l^a, l^b\} \mid z_{m_{g-1}}^{g-1})\, \bar{Q}_1^g(\{l^a, l^b\} \mid \tilde{z}_{m_{g-1}}^{g-1})^{\mathbb{1}[g\neq 1]}}{\mathcal{N}_{g-1}(\{l^a, l^b\})},$$

and

$$D^g(k) = C^g(\{k, k\}) + \sum_{l=1}^{K} C^g(\{k, l\}).$$

Then, we can write the $\mathcal{N}$ function more compactly, as follows:

$$\begin{aligned}
\mathcal{N}_g(\{k^a, k^b\}) &= v_{1,\{k^a,k^b\}}^g (2 - \delta_{k^a,k^b}) \sum_{\{l^a,l^b\}} C^g(\{l^a, l^b\}) + u_1^g\, C^g(\{k^a, k^b\}) \\
&\quad + \frac{w_{1,k^a}^g}{1 + \delta_{k^a,k^b}} \sum_{\{l^a,l^b\}} C^g(\{l^a, l^b\}) \left(\delta_{k^b,l^a} + \delta_{k^b,l^b}\right) \\
&\quad + \frac{w_{1,k^b}^g}{1 + \delta_{k^a,k^b}} \sum_{\{l^a,l^b\}} C^g(\{l^a, l^b\}) \left(\delta_{k^a,l^a} + \delta_{k^a,l^b}\right) \\
&= (2 - \delta_{k^a,k^b}) v_{1,\{k^a,k^b\}}^g \sum_{\{l^a,l^b\}} C^g(\{l^a, l^b\}) + u_1^g\, C^g(\{k^a, k^b\}) \\
&\quad + \frac{w_{1,k^a}^g D^g(k^b) + w_{1,k^b}^g D^g(k^a)}{1 + \delta_{k^a,k^b}}.
\end{aligned}$$

□

Proof of Proposition 6. We proceed as in the proof of Proposition 4, using the notation developed in the proof of Proposition 5. By marginalizing over the remaining $m_g - 1$ components, it turns out that the marginal distribution of $\tilde{Z}_1^g$ is:

$$\mathbb{P}\left[\tilde{Z}_1^g = \{l^a, k^b\} \mid z^{-g}, \tilde{z}^{1:(g-1)}\right] \propto \frac{\bar{Q}_1^g(\{k^a, k^b\} \mid z_{m_{g-1}}^{g-1})\, \bar{Q}_1^g(\{k^a, k^b\} \mid \tilde{z}_{m_{g-1}}^{g-1})^{\mathbb{1}[g\neq 1]}}{\mathcal{N}_{g-1}(\{k^a, k^b\})} V_1^g(z_1^{g+1} \mid \{k^a, k^b\}).$$

Since we have already obtained $V_1^g(z_1^{g+1} \mid \{k^a, k^b\})$, by computing the $\mathcal{N}$ function, sampling $\tilde{Z}_1^g$ can be performed easily, in $\mathcal{O}(K^2)$ time. Given $\tilde{Z}_1^g$, we proceed to sample each $\tilde{Z}_j^g$ sequentially, from $j = 2$ to $j = m_g$. It is easy to verify that, at the j-th step, we sample $\tilde{Z}_j^g$ from:

$$\mathbb{P}\left[\tilde{Z}_j^g = \{k^a, k^b\} \mid z^{-g}, \tilde{z}^{1:(g-1)}\right] \propto \bar{Q}_j^g(\{k^a, k^b\} \mid \tilde{z}_{j-1}^g)\, V_j^g(z_1^{g+1} \mid \{k^a, k^b\}).$$

Again, this can be easily performed in $\mathcal{O}(K^2)$ time, for each j. □

Proof of Proposition 7. The result for $F_1(k)$ is immediate from (7). For $j \in \{2, \ldots, p\}$,

$$\frac{F_{j+1}(k)}{f_{j+1}(x_{j+1} \mid k)} = \sum_{l=1}^{K} Q_{j+1}(k \mid l)\, F_j(l) = a_{j+1,k} \sum_{l=1}^{K} F_j(l) + b_{j+1} F_j(k).$$

□

Proof of Proposition 8. The case if $F_1(\{k^a, k^b\})$ follows directly from (8). For $j \in \{2, \ldots, p\}$,

$$
\begin{aligned}
\frac{F_{j+1}(\{k^a, k^b\})}{f_{j+1}(z_{j+1} \mid \{k^a, k^b\})} &= \sum_{\{l^a, l^b\}} \bar{Q}_{j+1}(\{k^a, k^b\} \mid \{l^a, l^b\})\, F_j(\{l^a, l^b\}) \\
&= a_{j+1,k^a} a_{j+1,k^b} (2 - \delta_{k^a,k^b}) \sum_{\{l^a, l^b\}} F_j(\{l^a, l^b\}) + (b_{j+1})^2 F_j(\{k^a, k^b\}) \\
&\quad + b_{j+1} \frac{a_{j+1,k^a}}{1 + \delta_{k^a,k^b}} \sum_{\{l^a, l^b\}} \left(\delta_{k^b,l^a} + \delta_{k^b,l^b}\right) F_j(\{l^a, l^b\}) \\
&\quad + b_{j+1} \frac{a_{j+1,k^b}}{1 + \delta_{k^a,k^b}} \sum_{\{l^a, l^b\}} \left(\delta_{k^a,l^a} + \delta_{k^a,l^b}\right) F_j(\{l^a, l^b\}) \\
&= a_{j+1,k^a} a_{j+1,k^b} (2 - \delta_{k^a,k^b}) \sum_{\{l^a, l^b\}} F_j(\{l^a, l^b\}) + (b_{j+1})^2 F_j(\{k^a, k^b\}) \\
&\quad + b_{j+1} \frac{a_{j+1,k^a}}{1 + \delta_{k^a,k^b}} \left(F_j(\{k^b, k^b\}) + \sum_{l=1}^{K} F_j(\{l, k^b\}) \right) \\
&\quad + b_{j+1} \frac{a_{j+1,k^b}}{1 + \delta_{k^a,k^b}} \left(F_j(\{k^a, k^a\}) + \sum_{l=1}^{K} F_j(\{l, k^a\}) \right).
\end{aligned}
$$

□

SUPPLEMENTARY NOTE 1: GOODNESS-OF-FIT OF THE HMM

We fit the fastPHASE HMM to the phased haplotypes for the 350k individuals in the UK Biobank retained in our analysis. The parameters of 22 separate models are estimated applying fastPHASE separately within each autosome. The flexibility of the HMM is controlled by the number K of haplotype motifs, which is important for the performance of *KnockoffZoom*. If K is too small, the knockoffs may not have the right LD structure to serve as negative controls, resulting in an excess of false discoveries; if K is too large, over-fitting may render our procedure overly conservative and reduce power. In order to choose a good value of K, we consider a wide range of alternatives and evaluate the goodness-of-fit of each model in terms of its ability to correctly predict missing values in a hold-out sample. For this purpose we divide the individuals into a training set of size 349,119 and a validation set of size 10,000; then, we mask 50% of the haplotypes from the second set and use the fitted HMMs to reconstruct their value given the observed data. The goodness-of-fit is thus measured in terms of imputation error: lower values indicate a more accurate model. The results corresponding to chromosome 22 are shown in Supplementary Table 22. Even though $K = 100$ seems optimal according to this metric, little improvement is observed above 50. Therefore, we choose $K = 50$ to generate knockoffs for the rest of the analysis, in the interest of computational speed. Finally, we verify that the goodness-of-fit of this HMM with $K = 50$ is significantly better than that of a multivariate Gaussian approximation of the genotype distribution,[15] with parameters estimated on all 359k samples, which has imputation error equal to 5.52%.

SUPPLEMENTARY NOTE 2: EXCHANGEABILITY DIAGNOSTICS

Having generated the knockoffs with the estimated HMM discussed above, it is interesting to verify their exchangeability with the real data. In theory, if the distribution of genotypes followed this HMM exactly, the joint distribution of $(\mathbf{X}, \tilde{\mathbf{X}})$ would be unchanged when $\{X_j : j \in G\}$ is swapped with $\{\tilde{X}_j : j \in G\}$, for any group $G \in \mathcal{G}$, by construction of $\tilde{\mathbf{X}}$. However, since our HMM can only approximate the true distribution of genotypes in the UK Biobank, this exchangeability is not perfect in practice. A simple way to quantify and visualize this exchangeability is to compute the covariance of the augmented matrix of explanatory variables $[\mathbf{X}, \tilde{\mathbf{X}}] \in \mathbb{R}^{n \times (2p)}$. For instance, we compare in the left-hand-side of Supplementary Figure 2 $r^2(X_j, X_k)$ with $r^2(\tilde{X}_j, \tilde{X}_k)$, for SNPs j, k in different groups on chromosome 22. In the right-hand-side of Supplementary Figure 2, we also compare $r^2(X_j, X_k)$ with $r^2(X_j, \tilde{X}_k)$, for the same pairs of SNPs. In both scatter plots, the points would concentrate around the 45° line if the HMM were exact.

We observe that the desired exchangeability holds approximately but some deviations occur, especially for lower-frequency variants (see Supplementary Figure 3), and when the knockoffs are constructed using low-resolution partitions. This should not be very surprising. First, empirical correlations involving lower-frequency variants are naturally noisier, and it is natural that the HMM fits better the distribution of more common variants. Second, the accuracy of knockoffs at low resolution depends on our ability to capture long-range correlations, which are generally weaker compared to short-range LD, but also slightly underestimated by the current implementation of our HMM. In fact, long-range correlations may be partially due to some underlying population structure that we are not explicitly trying to model.

The methods presented in this paper can naturally accommodate a more flexible implementation of the HMM that describes long-range correlations and population structure accurately; this extension will be presented soon in a separate work. For the time being, we have verified that the knockoffs described here lead to FDR control in practice, across a variety of numerical simulations involving the same real genotypes used in the analysis of the unrelated British individuals in the UK Biobank. Finally, we have verified that *KnockoffZoom* tends to select more common variants for all real phenotypes analyzed in this paper (Supplementary Figure 13); thus it does not seem to be practically affected by the lower accuracy of knockoffs for rarer variants. However, it may be of particular interest to focus on studying rarer variants in future applications. Therefore, we are also working to improve the current algorithm used to estimate the HMM parameters in order to model their distribution more closely.

SUPPLEMENTARY NOTE 3: COMPUTATIONAL AND MEMORY RESOURCES

The computation time required by *KnockoffZoom* for the data analysis in this paper is reported in Supplementary Table 2, for each operation described in the flowchart of Supplementary Figure 4. The entire procedure, starting from phased haplotypes, takes about 12 days—this is less than the time needed for phasing.[5] In principle, our procedure can be applied to unphased genotypes, although this is not recommended if the dataset is very large, for the knockoff generation would be slower. If we want to analyze different phenotypes, only the fourth and fifth modules need to be repeated (the latter requires negligible resources). The analysis of a new trait for the same individuals in the UK Biobank takes us less than 12 hours with an efficient implementation of the fourth module. As a comparison, BOLT-LMM takes between a few days and a week, depending on the phenotype. These are rough upper bounds because the exact time depends on the computing hardware and other factors.

The resource requirements of our method are also summarized in Supplementary Table 2. The computations in modules 1–3 are divided between 22 machines, one for each autosome (our estimates refer to the longest one). The memory footprint is low, except for clustering, which requires about 200GB. If this becomes limiting, one can define the LD blocks differently, e.g., locally and concatenating the results. By comparison, BOLT-LMM requires approximately 100 GB with the same data.

We can easily analyze the theoretical complexity of the first and third modules. In order to efficiently fit an HMM for the genotypes, the haplotypes are phased using SHAPEIT3;[5] then fastPHASE[3] is applied to the inferred haplotypes. The computational complexity of SHAPEIT3 and fastPHASE are $\mathcal{O}(pn \log n)$ and $\mathcal{O}(pnK)$, respectively, where p is the number of variants, n is the number of individuals and K is the number of haplotype motifs in the HMM. Our analytical calculations for the fastPHASE HMM have reduced the cost of the third module to $\mathcal{O}(pnK)$. If the phased haplotypes are not available, a modified version of our algorithm has cost $\mathcal{O}(pnK^2)$. The second and fourth modules are more difficult to analyze theoretically, as different choices of tools are available to cluster the variants and compute the test statistics. For the latter, we rely on very fast and memory-efficient implementations of sparse linear and logistic regression.[13] The fifth and final module of *KnockoffZoom* is computationally negligible.

SUPPLEMENTARY NOTE 4: COORDINATING MULTI-RESOLUTION DISCOVERIES

Here, we show how to combine the tests statistics computed by our method at different resolutions in order to coordinate the discoveries, so that no "floating" blocks are reported, while rigorously controlling the FDR.

Let $r = 1, \dots, R$ index the resolutions we consider (Methods), ordered from the lowest to the highest. At each resolution r, the p variants are partitioned into L^r groups: $\mathcal{G}^r = (G^r_1, \dots, G^r_{L^r})$. By construction, the partitions are nested, so that for each resolution $r > 1$ and each group $g \in \{1, \dots, L^r\}$ there is a unique parent group $\text{Pa}(g, r) \in \{1, \dots, L^{r-1}\}$ such that $G^r_g \subseteq G^{r-1}_{\text{Pa}(g,r)}$. Suppose that we have computed the test statistics $\mathbf{W}^r = \{W^r_g\}_{g=1,\dots,L^r}$ at each resolution (Methods). To avoid "floating" discoveries, we can only select groups whose parent has been discovered at the resolution below. We can enforce this consistency property while preserving an FDR guarantee at each resolution by slightly modifying the final filtering step that computes the significance thresholds (Methods). Instead of applying the knockoff filter separately at each resolution, we proceed with Algorithm 5 sequentially, from lower to higher resolutions.

Algorithm 5 Consistent-layers knockoff filter

Input: Partitions $\mathcal{G}^r$, for each resolution level $r \in \{1, \dots, R\}$;

Test statistics $\mathbf{W}^r = (W^r_1, \dots, W^r_{L^r})$, for each resolution level $r \in \{1, \dots, R\}$;

FDR target level q.

Compute t^{*1} applying the usual knockoff filter, at nominal level $q/1.93$, to W^1:

$$t^{*1} = \min \left\{ t^1 \geq 0 : \frac{1 + |\{g : W^1_g \leq -t^1\}|}{|\{g : W^1_g \geq t^1\}|} \leq \frac{q}{1.93} \right\}.$$

Select discoveries at the lowest resolution: $\hat{S}^1 = \{g : W^1_g \geq t^{*1}\}$.

for $r = 2$ to $r = R$ **do**

Compute t^{*r} as follows:

$$t^{*r} = \min \left\{ t^r \geq 0 : \frac{1 + |\{g : W^r_g \leq -t^r\}|}{|\{g : W^r_g \geq t^r,\ \text{Pa}(g, r) \in \hat{S}^{r-1}\}|} \leq \frac{q}{1.93} \right\}.$$

Select discoveries at this resolution: $\hat{S}^r = \{g : W^r_g \geq t^{*r},\ \text{Pa}(g, r) \in \hat{S}^{r-1}\}$.

We prove in Proposition 9 that the FDR is controlled at each resolution by Algorithm 5, which is closely inspired by previous work.[1] The correction of the FDR level by the factor 1.93 is required in the proof for technical reasons, although we have observed in numerical simulations that this may be practically unnecessary; see Supplementary Figure 5 for empirical evidence. Therefore, to avoid an unjustified power loss while retaining provable guarantees, we have not reported the

results obtained with Algorithm 5 in the main paper. However, we have applied it on real data and reported the results in Supplementary Figure 5. Based on our experience, should "floating" discoveries be particularly undesirable, we believe that it may be safe to apply Algorithm 5 even without the 1.93 factor.

Proposition 9. *Denote by* FDR^r *the FDR at resolution* r *for the discoveries obtained with Algorithm 5. If the data sampling assumptions (Methods) are valid and the knockoff exchangeability holds (Methods), then* $\mathrm{FDR}^r \leq 1.93 \cdot q$, $\forall r \in \{1, \ldots, R\}$.

Proof. It was shown in earlier work[1] (proof of Theorem 1 therein) that

$$\mathbb{E}\left[\sup_{t^r \geq 0} \frac{|\{g : W_g^r \geq t^r\} \cap \mathcal{H}_0^r|}{1 + |\{g : W_g^r \leq -t^r\}|}\right] \leq 1.93,$$

where $\mathcal{H}_0^r \subseteq \{1, \ldots, L^r\}$ is the set of null groups at resolution r. Therefore,

$$\begin{aligned}
\mathrm{FDR}^r = \mathbb{E}\left[\frac{|\hat{S}^r \cap \mathcal{H}_0^r|}{|\hat{S}^r|}\right] &\leq \mathbb{E}\left[\frac{|\{g : W_g^r \geq t^{*r}\} \cap \mathcal{H}_0^r|}{|\hat{S}^r|}\right] \\
&= \mathbb{E}\left[\frac{|\{g : W_g^r \geq t^{*r}\} \cap \mathcal{H}_0^r|}{1 + |\{g : W_g^r \leq -t^{*r}\}|} \cdot \frac{1 + |\{g : W_g^r \leq -t^{*r}\}|}{|\hat{S}^r|}\right] \\
&\leq \frac{q}{1.93} \cdot \mathbb{E}\left[\frac{|\{g : W_g^r \geq t^{*r}\} \cap \mathcal{H}_0^r|}{1 + |\{g : W_g^r \leq -t^{*r}\}|}\right] \\
&\leq \frac{q}{1.93} \cdot \mathbb{E}\left[\sup_{t^r \geq 0} \frac{|\{g : W_g^r \geq t^r\} \cap \mathcal{H}_0^r|}{1 + |\{g : W_g^r \leq -t^r\}|}\right] \\
&\leq q.
\end{aligned}$$

□

SUPPLEMENTARY NOTE 5: ASSESSING INDIVIDUAL SIGNIFICANCE

In addition to controlling the FDR, we can quantify, to some extent, the statistical significance of individual findings. We have not discussed this in the paper in the interest of space and because the underlying theory is not fully developed. Nonetheless, the main idea is intuitive. Recall that the basic ingredient of the knockoff filter[16] is a suitable estimate of the false discovery proportion: FDP(t), i.e., the fraction of false discoveries if we reject all hypotheses in $\{g : W_g \geq t\}$ (the test statistics W_g are defined in the Methods section). Additional information can be extracted from the test statistics by estimating a local version of the FDR[17], e.g., the fraction of false discoveries in $\{g : t - \Delta t_1 \leq W_g \leq t + \Delta t_2\}$, for some choice of Δt_1 and Δt_2. For this purpose, we estimate:

$$\widehat{\mathrm{Fdp}}(t, \Delta t_1, \Delta t_2) = \frac{c + |\{g : -(t + \Delta t_2) \leq W_g \leq \min(0, -t + \Delta t_1)\}|}{\max(1, |\{g : \max(0, t - \Delta t_1) \leq W_g \leq t + \Delta t_2\}|)}, \tag{10}$$

with either $c = 0$ (more liberal) or $c = 1$ (more conservative). The usual estimate of the FDP computed by the knockoff filter[16] is recovered if $\Delta t_1 = 0$ and $\Delta t_2 \to \infty$:

$$\widehat{\mathrm{FDP}}(t) = \frac{c + |\{g : W_g \leq -t\}|}{\max(1, |\{g : W_g \geq t\}|)},$$

Intuitively, findings with low $\widehat{\mathrm{Fdp}}$ should be less likely to be false positives. The choices of Δt_1 and Δt_2 determine the accuracy of our estimates: if the span is smaller, the local FDR encodes information at higher resolution; however, our estimate is noisier because it relies on fewer statistics. We evaluate (10) in simulations (Supplementary Figure 14) and on real data (Supplementary Figure 15), setting $\Delta t_1 = 100 = \Delta t_2$ and $c = 0$ (we use $c = 1$ in the knockoff filter). This approach is reasonable if *KnockoffZoom* reports sufficiently many discoveries; otherwise, the estimated local FDP may have high variance.

The simulation in Supplementary Figure 14 is carried out on an artificial phenotype with the first genetic architecture in Supplementary Table 3 and heritability $h^2_{\text{causal}} = 0.7$. We plot the FDP as a function of the number of selected variants, in the order defined by the test statistics.

We highlight two observations from these results. First, the estimated cumulative FDP tracks the true FDP closely, consistently with the fact that the FDP of *KnockoffZoom* is usually below the nominal FDR level in simulations. The low variance in the estimated FDP may be explained by the size of the dataset and the large number of discoveries. Second, the estimated local FDP also approximates the corresponding true quantity quite precisely, especially for the statistics above the rejection threshold. This may be very valuable in practice, because it provides us with a good educated guess of which discoveries are likely to be false positives. Whether we can rigorously state more precise results is an interesting question that we plan to explore deeper in the future.

SUPPLEMENTARY NOTE 6: MISSING AND IMPUTED VARIANTS

We have not included imputed variants in our analysis because they do not contain any information on the trait of interest, in addition to that carried by the genotyped variants. Since this statement appears to clash with common practice, we discuss it here.

Note that imputed genotypes are not directly observed; instead, their values are set on the basis of the observed genotypes and models for linkage disequilibrium derived from other datasets. The imputation process may not be deterministic—there is some randomness in the reconstruction of the imputed genotypes—but this extra stochasticity is independent of the phenotype and hence uninformative. Even if the imputation is very accurate, the following fundamental reality does not change: the imputed genotypes are not observed, they are reconstructed entirely on the basis of the observed ones. Therefore, imputed genotypes cannot provide meaningful additional information to that already carried by the genotyped variants. Why then has the field found them useful?

Imputed genotypes may help can increase the power to detect associated loci, as a result of the mismatch between the models used to analyze the relation between genotypes and phenotypes (typically linear) and the true biological mechanism (unknown). To illustrate, consider an example in the context of the standard analysis of GWAS data, where a univariate test probes for *marginal* association between a phenotype and each variant. Consider the case where an untyped variant B can be imputed with high accuracy, using the genotypes of two neighboring variants A and C (in the interest of concreteness, let's say that the minor allele of B is usually observed only in conjunction with a specific haplotype of A and C). It is quite possible that the association test between the imputed variant B and the phenotype rightly results in a substantially smaller p-value than the association tests between the phenotype and either of the two variants A and C whose genotypes are used for imputation. This could happen because B is causal, and each of the genotyped variants only provides an imperfect proxy. Or it could happen because the phenotype depends on the two observed variants A and C in a nonlinear fashion: one needs the two alleles that define the haplotype corresponding to the minor allele of B to observe a change in the phenotype mean. The two situations are indistinguishable on the basis of data on A and C alone. However, this is not a problem if we simply want to identify the presence of an association. As long as we interpret a significant p-value as indicating the presence of an associated locus, without describing causal mechanisms, the increase in power is all that matters.

The situation changes substantially if we move the target from identifying a locus that contains causal variants to pinpointing the causal variants. In the first situation described above, B is causal,

and changing the value of its allele would result in a change of the expected value of the phenotype. In the second situation, changing the major allele of B to its minor form, without touching the alleles at the neighboring variants A and C, will result in no change on the expected value of the phenotype. Distinguishing between these two situations on the basis of imputed data alone is not possible: any algorithm that would report $\{B\}$ or $\{A, C\}$ separately (as opposed to $\{A, B, C\}$) as containing the causal variants would do so either by relying on extra assumptions (e.g. causal sets contain the smallest possible number of variables or the true model is linear), or arbitrarily. Only genotyping B, and observing the few cases in which its minor allele occurs without the specific haplotype of $\{A, C\}$ used for imputation, could allow one to choose between the two causal models. To put these issues in a broader perspective, identifying causal mechanisms without relying on experiments is notoriously difficult (recall the refrain "correlation is not causation"). Getting a handle on causal effects from large-scale observational data is one of the contemporary challenges in statistics and data science. From this perspective, the attempt to attribute causal effects to variables that are not even observed, let alone modified in an experiment, seems quite audacious. This underscores the danger of attributing causal effects to imputed variants.

The inferential framework of *KnockoffZoom* naturally steers the researchers away from making misleading causal statements on imputed variants. By construction, the imputed genotypes are independent of the phenotype after conditioning on the genotyped variants, and all the conditional hypotheses involving imputed variants are null. The situation is different for other fine-mapping tools (e.g., CAVIAR or SUSIE), even if these also investigate "conditional" hypotheses that probe the role of a variant given the other SNPs. These methods analyze several variants in the same locus using a Bayesian multivariate regression model in which the genetic design matrix $\mathbf{X}$ is considered fixed. Therefore, it is not meaningful to ask about conditional independence between the trait Y and $\mathbf{X}$, since the latter is not random. Instead, in this context, "conditional" testing corresponds to asking which coefficients are nonzero in the assumed multivariate linear model for $Y \mid \mathbf{X}$. Once $\mathbf{X}$ is considered fixed, there is no technical difference between a measured variant and an imputed one: the machinery will take them all as input and treat them equally.

Nevertheless, the same conceptual difficulties in establishing causal models on the basis of imputed variants discussed above still apply. On the one hand, if the imputation quality is low, one ends up analyzing variants that do not exist in reality, so the possible discoveries would not be scientifically relevant. On the other hand, high-quality imputation is only possible if the genotyped variants explain almost all of the variation in the missing variants. If this dependence is linear, the genotyped and imputed variants may be highly collinear and testing in the multivariate linear

model powerless. This lack of power may be mitigated if the imputation process is highly nonlinear, since linear models are only sensitive to linear dependencies among variables. However, trusting the scientific validity of these discoveries would require one to place a lot of faith in the correctness of the linear model, as explained above.

While the conditional testing framework of *KnockoffZoom* intrinsically acknowledges the impossibility of differentiating between the effect of a missing variant from that of the genotyped ones used to impute it, two observations that have motivated practitioners to look at imputed variants continue to remain valid: (1) not all genetic variation is genotyped and therefore we need to consider the possibility that causal variants are untyped; (2) imputed variants can be utilized to construct more powerful tests. We now discuss how we can leverage these in our framework.

First, we have been careful in defining and interpreting our hypotheses so as to be meaningful in a context where not all variants are typed (1). This is why we choose to test spatially contiguous blocks of genotyped variants for association and report genomic intervals spanned by these blocks. This way, if *KnockoffZoom* returns a significant group of genotyped variants, we can be reasonably sure that either one of these variants is causal or there is at least one untyped causal variant in the corresponding genomic interval. While the *KnockoffZoom* methodology is agnostic to the choice of variable grouping and therefore can be applied to non-contiguous groups, such groups would be more difficult to interpret as long as there are untyped variants. For these reasons, we also find it most meaningful to interpret the "size" of a knockoff discovery as the width of the genomic interval it spans, rather than the number of genotyped variants it contains.

Second, while the resolution of the discoveries is fundamentally limited by the genotyped data, we remark that it is possible to leverage imputed variants with *KnockoffZoom* to increase power (2). Even if we are testing hypotheses defined only in terms of genotyped variants, we can use test statistics that capitalize on imputed ones, so as to most effectively capture the signal associated with a group of genotyped variants. We leave the implementation of such an approach for future work.

SUPPLEMENTARY NOTE 7: OTHER FINE-MAPPING METHODS

CAVIAR represents a broader class of popular tools, including FINEMAP,[19] PAINTOR,[20] and many others[21] that we do not consider explicitly here. It has recently been pointed out that these procedures cannot distinguish between multiple causal variants within the genomic region under analysis,[22] and SUSIE was proposed to overcome this limitation. SUSIE is based on Bayesian stepwise regression and uses the original data instead of relying on summary statistics.[23] However, neither CAVIAR nor SUSIE are designed to be applied genome-wide for the analysis of complex traits due to computational and statistical reasons, since they rely on relatively simple models with a small number of distinct causal effects. Therefore, we apply them locus-by-locus in a two-step procedure starting with BOLT-LMM.

The two-step procedure is calibrated to target a nominal error rate that is comparable to the FDR of *KnockoffZoom*. Regarding SUSIE, we simply apply it with nominal coverage parameter equal to 90%, so that at most 10% of its reported findings are expected to be false positives. In the case of CAVIAR, the solution is similar but it requires a more careful explanation. Under certain modeling assumptions, CAVIAR is designed to control the probability that any causal variants are erroneously discarded from the input candidate set[23] (but it does not tell apart distinct causal effects). Therefore, assuming that the input always contains at least one causal SNP (as we ensure in our simulations by applying BOLT-LMM with aggressive clumping), CAVIAR indirectly targets, within the two-step procedure, a notion of FDR similar to that of SUSIE and *KnockoffZoom*. In particular, setting the control parameter of CAVIAR equal to 90%, we can expect that at most 10% of the reported findings do not contain at least one causal variant.

SUPPLEMENTARY NOTE 8: IMPLEMENTATION OF ALTERNATIVE METHODS

The code used to run the comparisons discussed in this paper is public (`https://github.com/msesia/ukbiobank_knockoffs`), in addition to the code that implements *KnockoffZoom*. The version numbers and the relevant parameters of all third-party software are summarized below.

- BOLT-LMM. We apply BOLT-LMM (v. 2.3.2) using the default settings recommended in the official user manual (`https://data.broadinstitute.org/alkesgroup/BOLT-LMM/`). In the numerical experiments, we fit the model parameters on a subset of SNPs to reduce the computation cost (as suggested in the user manual for the analysis of large data sets), while we use all SNPs to fit the model parameters in the real data analysis.

- PLINK. We clump nearby variants identified by BOLT-LMM by applying PLINK (v. 1.90) using a 5 Mb window and an r^2 threshold equal to 0.01 (to include sub-threshold SNPs in LD with an existing clump), while the secondary significance level is 10^{-2}, as in earlier work.[18]

- CAVIAR. We perform fine-mapping using CAVIAR (v. 2.2) with confidence level equal to 0.9, setting the maximum number of causal SNPs equal to 2 to keep the computational cost manageable in large loci. The other parameters are equal to their default values.

- SUSIE. We apply the SUSIE R package (v. 0.7.1) with nominal coverage 0.9, setting the scaled prior variance parameter equal to 0.1 (this performed better in our examples compared to the default value of 0.2) and allowing the estimation of the residual variance. The other parameters are equal to their default values. Note that the output of SUSIE needs to be simplified because it occasionally reports overlapping subsets of SNPs; therefore, we consolidate those that are not disjoint and retain the smallest if one is included in the other.

- LMM ORACLE. The FDR oracle employed in our simulations is implemented as follows. Significant loci are clumped by applying the PLINK algorithm to the LMM p-values, over a discrete grid of possible values for the primary significance threshold. We use a wide logarithmic scale: 5×10^{-9}, 5×10^{-8}, 5×10^{-7}, 5×10^{-6}, 10^{-5}, 2×10^{-5}, 5×10^{-5}, 10^{-4}, 2×10^{-4}, 5×10^{-4}, 10^{-3}. The number of discoveries and the FDP are computed for each threshold and experiment. To mitigate the discretization, we interpolate the results between the grid points. Then, we count the true discoveries corresponding to the most liberal threshold that controls the FDP (or the FDR, if the experiments are replicated multiple times with the same parameters).

SUPPLEMENTARY NOTE 9: THE DIFFICULTY OF CONTROLLING THE FDR

Targeting the FDR in a GWAS is difficult; in particular, it is challenging to control it over distinct (conditional) discoveries using LMM (marginal) p-values. We illustrate the challenges by considering two variations of the Benjamini-Hochberg (BH) procedure.[24] First, we apply the BH correction to the BOLT-LMM p-values and report discoveries with the usual PLINK clumping. Second, we use BOLT-LMM to test pre-determined hypotheses defined over the *KnockoffZoom* LD blocks. For this purpose, we summarize the p-values corresponding to SNPs in the same block into a single number, a Simes p-value,[25] that we provide to the BH procedure without further clumping. For example, we consider two resolutions: 0.226 Mb or 0.018 Mb-wide blocks, on average.

The observed outcome is an excess of false positives, regardless of how we count distinct discoveries; see Supplementary Figure 6. With the first approach, the FDR is inflated because the BH procedure counts the discoveries *pre-clumping*, while the FDR is evaluated on findings that are defined differently, *post-clumping*.[26] Moreover, these p-values are only designed for marginal hypotheses, while we are interested in distinct (conditional) findings.[27] With the second approach, the FDR would be controlled if the p-values for loci in different groups were correct and independent. However, this is not the case, even when the blocks are large, because some inter-block LD remains. These examples are not exhaustive, but they illustrate the fundamental problem that the LMM p-values are not calibrated for conditional hypotheses, as already shown in the simulations.

A variation of the BH procedure has been recently proposed in combination with clumping,[26] but it does not address conditional hypotheses. An alternative solution is based on the distributions of scan statistics,[27] although we are not aware of whether this has been tested. A different approach based on penalized regression[28] has shown promise, but it relies on stringent modeling assumptions.

SUPPLEMENTARY NOTE 10: CONTIGUOUS GROUPS AND RESOLUTION

Until now, we have compared the fine-mapping resolution of different methods according to the average width spanned by each discovery, measured in base pairs. We believe this is a particularly meaningful measure of localization in SNP-array data sets, where the true "causal" variants may not have been genotyped, and discoveries should be interpreted as indicating a genetic region of interest (as precisely as possible) rather than exactly identifying the causal variants. However, it is also informative to look at different summary statistics of the discoveries to gain a broader perspective. For instance, we compare in Supplementary Figure 12 the average size of the discoveries, measured in number of SNPs, or their homogeneity, measured in terms of the average r^2 within each group (i.e., the average of the squared within-group correlation matrix over all entries), for the different methods in the same settings as in Figure 4 of the main text and in Supplementary Figure 11.

These results show that *KnockoffZoom* typically reports discoveries containing more SNPs compared to its alternatives, which is not very surprising given that it tests pre-defined (and in this case contiguous) hypotheses, and therefore lacks some of the flexibility of other fine-mapping tools to explicitly discard nearby variants that are not significant. However, these numbers become more similar as the signal strength increases and *KnockoffZoom* increasingly localizes causal effects at the highest resolution. Regarding the homogeneity of the discoveries, the SNPs reported within the same group by SUSIE are the most similar to each other, with an average r^2 approximately equal to 0.9. The homogeneity of the *KnockoffZoom* discoveries is typically lower compared to those of SUSIE but higher compared to those of CAVIAR. In any case, as the signal strength increases, *KnockoffZoom* increasingly reports single-SNP discoveries.

SUPPLEMENTARY NOTE 11: COMPUTING THE TEST STATISTICS

The feature importance measures that we have adopted to define the test statistics of *KnockoffZoom* are based on sparse multivariate linear and logistic regression, since Bayesian and penalized regression approaches are currently the state-of-the-art for predicting complex traits.[7,8] However, our methodology could easily incorporate other scalable machine learning tools, like SVMs, random forests, and deep learning.[9–11] In principle, the test statistics could also be computed using cheaper single-marker methods, e.g., by contrasting the univariate regression p-values for the original genotypes and the knockoffs; however, we do not recommend this for two reasons. First, our method is more powerful if combined with a multi-marker model that explains a larger fraction of the variance in the phenotypes. Second, multivariate methods are less susceptible to the confounding effect of population structure.[12] Therefore, they improve our robustness against possible violations of the modeling assumptions, i.e., the HMM approximation of the distribution of the genotypes.

In this paper, we use the R packages `bigstatsr` and `bigsnpr`[13] to fit a sparse generalized linear model the trait given the augmented matrix of explanatory variables $[\mathbf{Z}, \mathbf{X}, \tilde{\mathbf{X}}] \in \mathbb{R}^{n \times (m+2p)}$. Here, $\mathbf{Z}$ is a matrix containing the covariates for all individuals, while $\mathbf{X}$ and $\tilde{\mathbf{X}}$ indicate the observed genotypes and their knockoff copies, respectively. The regularization penalty is tuned using a modified form of 10-fold cross-validation. The regression coefficients for each variable are averaged over the 10 folds, finally obtaining an estimate of the quantity $\hat{\beta}_j(\lambda_{\text{CV}})$ from the Methods section. Since the knockoff-augmented genotype data is stored on an hard-drive as a memory-mapped file, the algorithm is memory efficient.[14] In addition, if the knockoffs in a group g are known in advance to have very little power as negative controls, e.g., $\max_{j \in G} r^2(X_j, \tilde{X}_j) > 0.99$, the corresponding hypothesis is automatically discarded by setting $W_g = 0$. This operation is independent of the phenotypes and preserves the symmetry required to control the FDR,[15] but it may improve power, especially at high resolution when the pairwise r^2 can be high (Supplementary Figure 1).

Even though the order in which the variables are provided to the black box that computes the feature importance measures (e.g., the lasso) does not matter in theory, it can make a difference in practice. For instance, numerical instabilities may induce the black box to give an unfair advantage to variables found earlier in the input, which would result in a symmetry break if the input is $[\mathbf{X}, \tilde{\mathbf{X}}]$, possibly leading to a loss of FDR control. For this reason, our implementation randomly swaps each pair of genotypes and knockoffs before computing the variable importance measures. The original identity of the knockoffs is only revealed later to determine the sign of the test statistics. Therefore, the FDR guarantee of *KnockoffZoom* is completely immune to such numerical instabilities.

SUPPLEMENTARY FIGURES

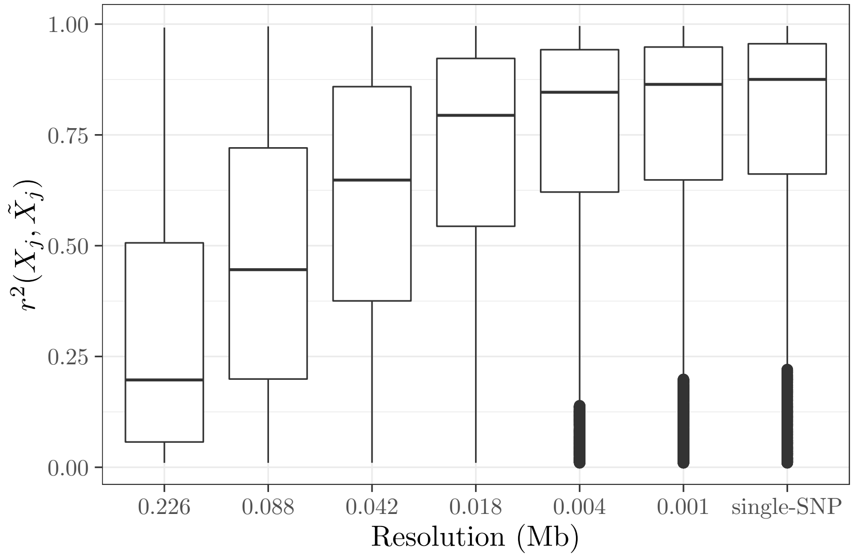

SUPPLEMENTARY FIGURE 1. **Knockoff power diagnostics.** Distribution of the average pairwise r^2 between genotypes and knockoffs at different levels of resolution, for 591,513 SNPs in the UK Biobank. Other details as in Supplementary Table 1.

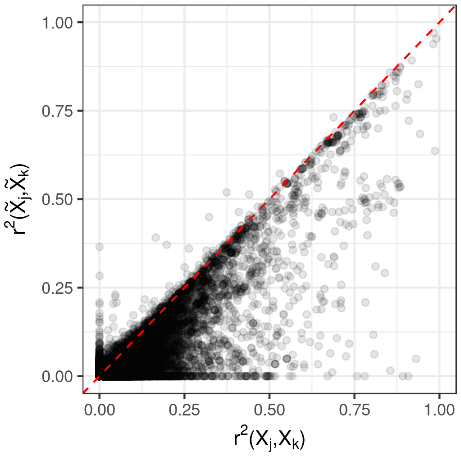

(a) 0.226 Mb resolution

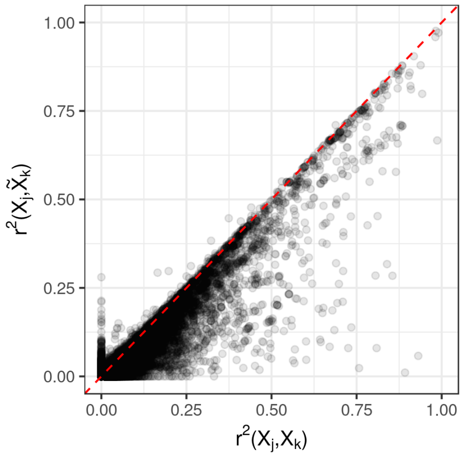

(b) 0.226 Mb resolution

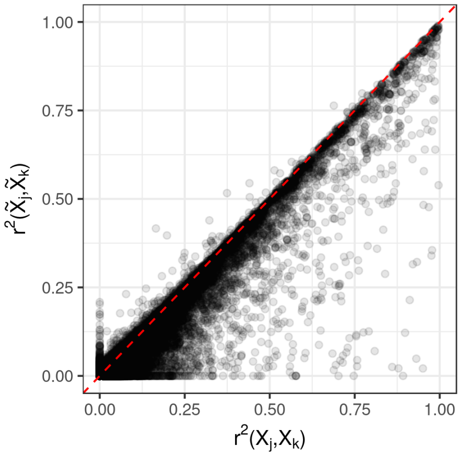

(c) 0.042 Mb resolution

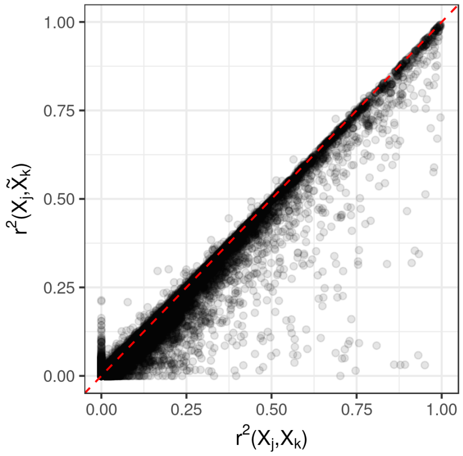

(d) 0.042 Mb resolution

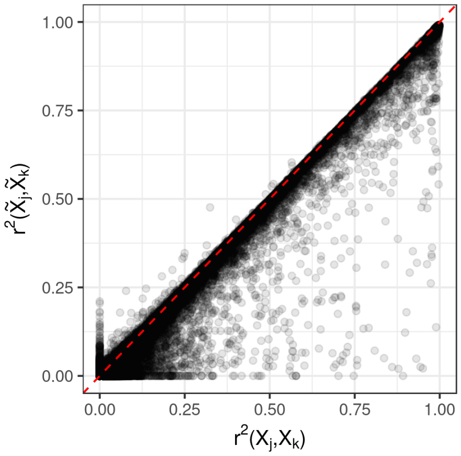

(e) single-SNP resolution

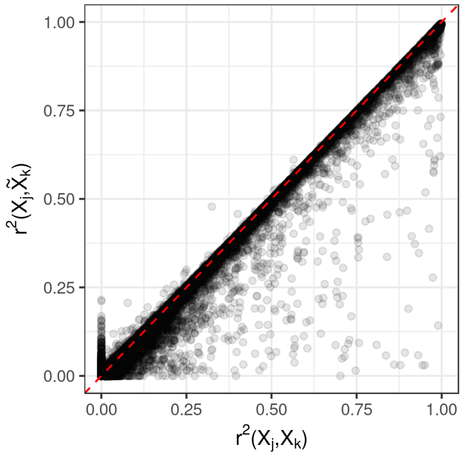

(f) single-SNP resolution

SUPPLEMENTARY FIGURE 2. **Knockoff exchangeability diagnostics.** Correlation exchangeability diagnostics for knockoffs at different resolutions. The definition of these diagnostics is discussed in the Supplementary Note 2.

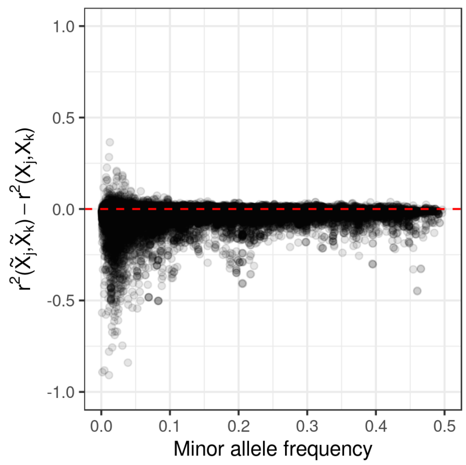

(a) 0.226 Mb resolution

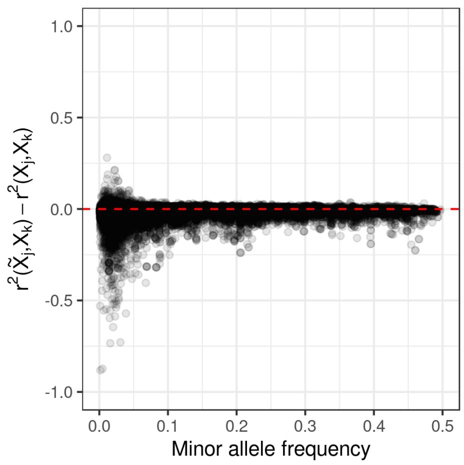

(b) 0.226 Mb resolution

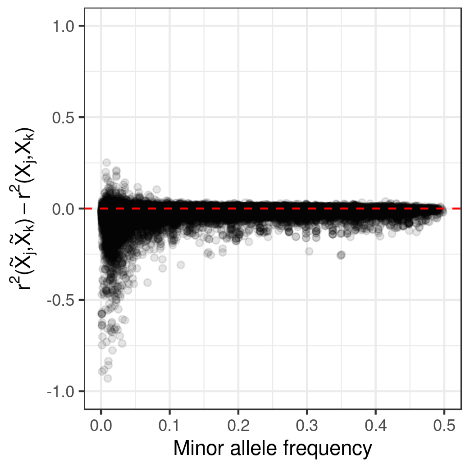

(c) 0.042 Mb resolution

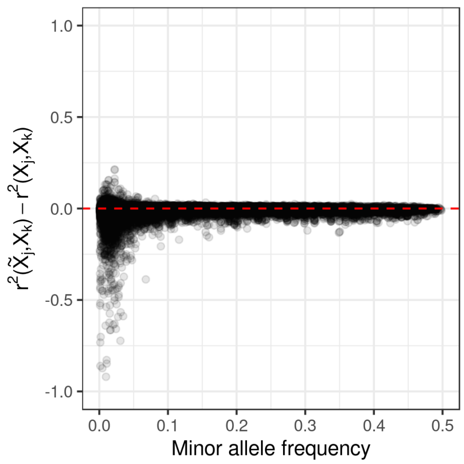

(d) 0.042 Mb resolution

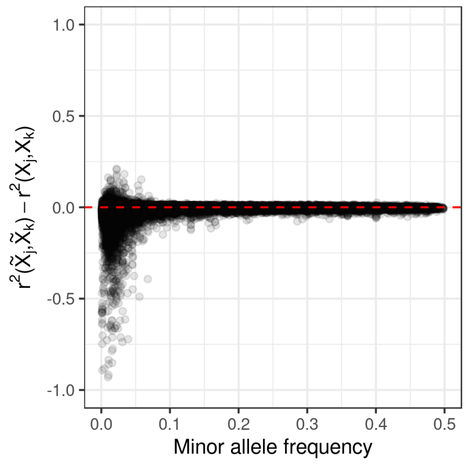

(e) single-SNP resolution

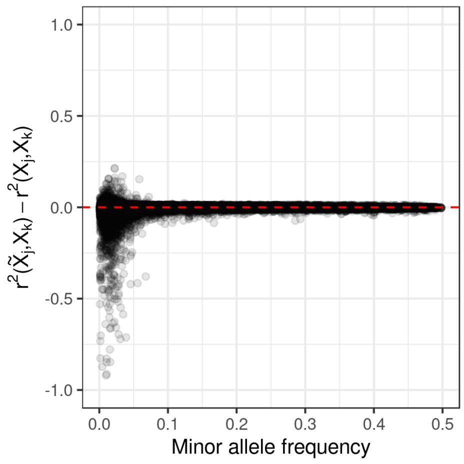

(f) single-SNP resolution

SUPPLEMENTARY FIGURE 3. **Exchangeability and allele frequency.** Deviations from their ideal values of the knockoff exchangeability diagnostics in Supplementary Figure 2, as a function of the smallest minor allele frequency for each pair of variants.

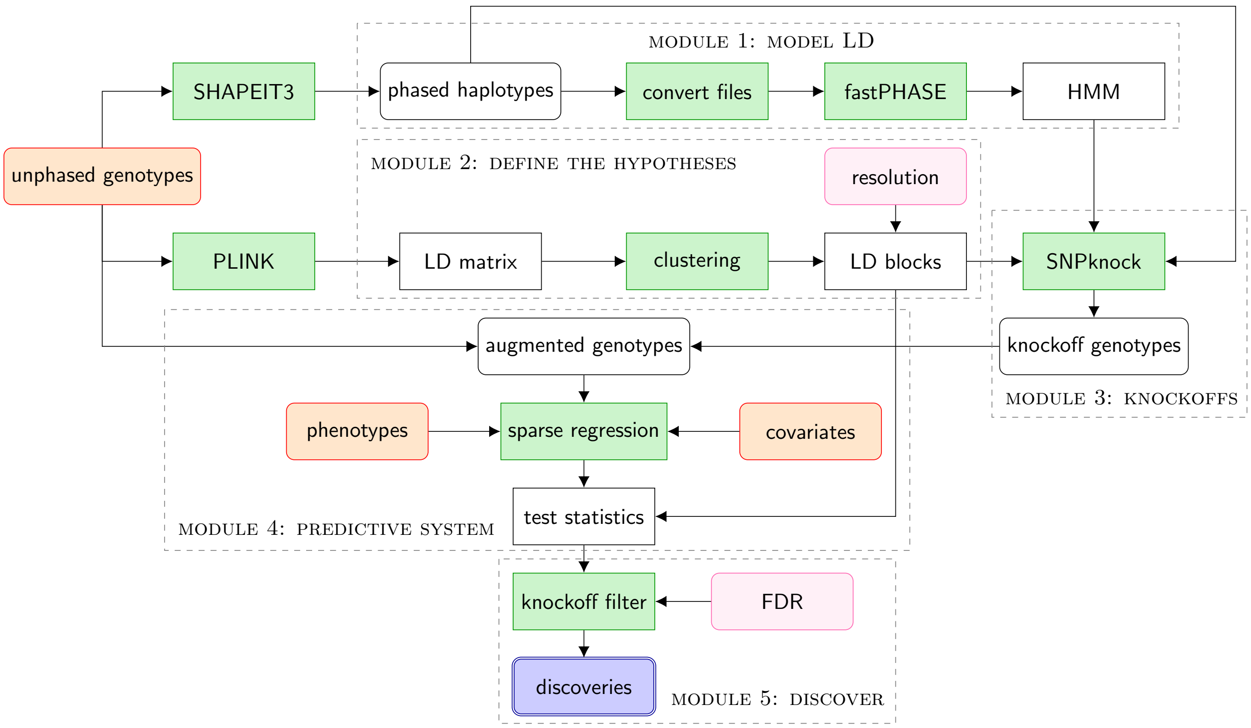

SUPPLEMENTARY FIGURE 4. **Method schematic.** Schematic of the *KnockoffZoom* method for the analysis of GWAS data. The inputs are the unphased genotypes (or the phased haplotypes, if available), the phenotypes, and any relevant covariates (e.g., age, sex, other demographics, principal components). The user chooses the resolution at which our method performs genome-wide fine-mapping and the nominal FDR level. In the first module, an HMM is fit using the phased haplotypes using fastPHASE. Meanwhile, the typed loci are assigned to blocks that represent our units of inference (second module), based on the observed LD, the physical locus positions and the resolution. Then, the estimated HMM and the partition of the variants are used by our software (SNPknock) to generate the knockoffs (third module). In the fourth module, a test statistic for each block is computed by a machine-learning system that predicts the phenotype and estimates the feature importance of genotypes and knockoffs, without knowing which one is which. The knockoffs serve as negative controls, allowing the filter to calibrate the statistics for FDR control (fifth module).

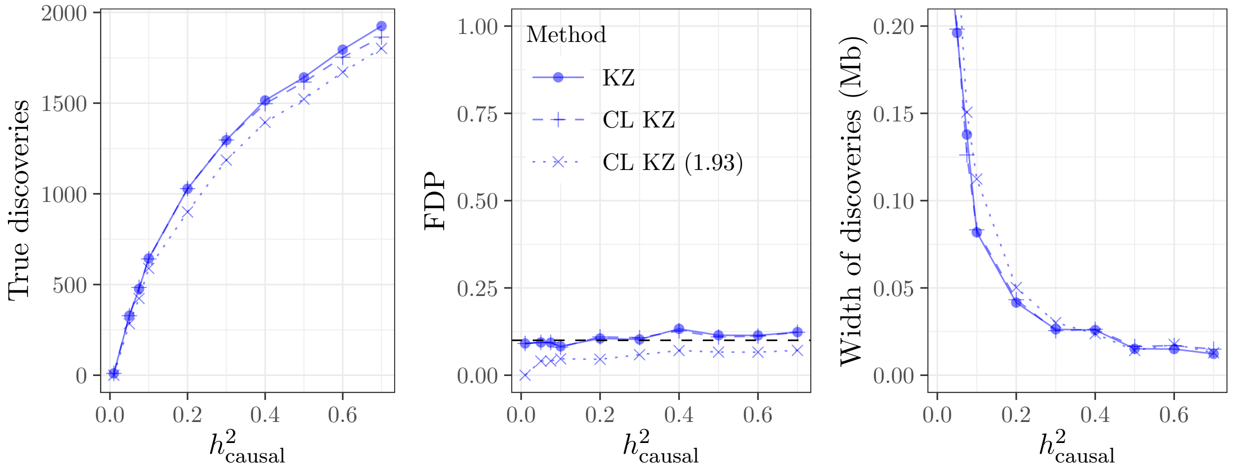

(a)

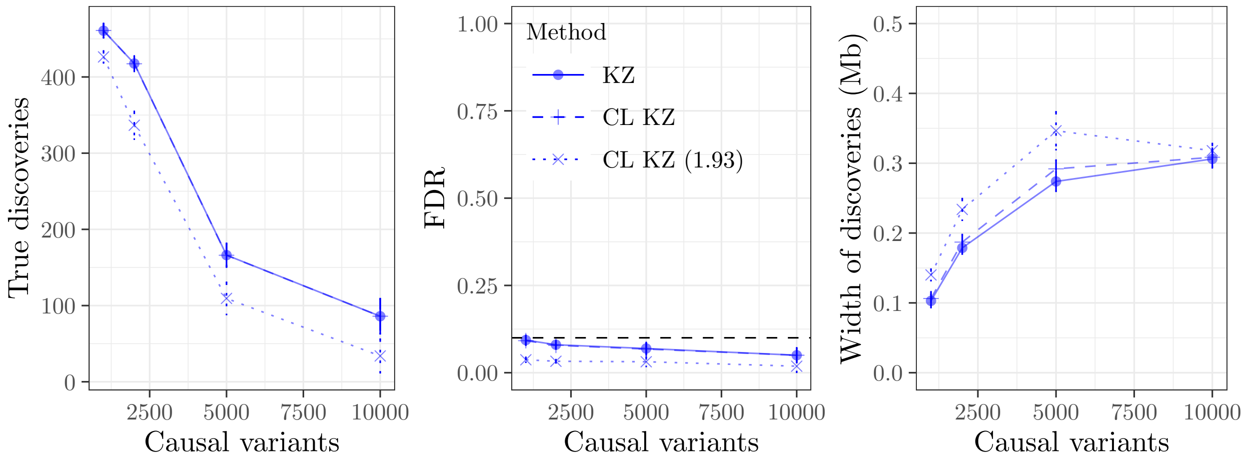

(b)

SUPPLEMENTARY FIGURE 5. **Simulations with coordinated discoveries.** Performance of *KnockoffZoom* (KZ) with and without the consistent-layers knockoff filter. When enforcing layer consistency, we either include or not include the 1.93 factor discussed in Supplementary Note 4; i.e., CL KZ (1.93), CL KZ. The curves corresponding to KZ and CL KZ are almost overlapping. The numbers of findings are simplified in all cases by counting only the finest discoveries in each locus, as in Figure 4 of the main text. (a): Other details as in Figure 4 of the main text. (b): Other details as in Supplementary Figure 8. These results indicate that the consistent-layers filter without the 1.93 correction factor performs similarly to the usual filter applied separately at each resolution, although it loses some power at high resolution. By contrast, the 1.93 factor is overly conservative, especially if there is a large number of causal variants with weak signals.

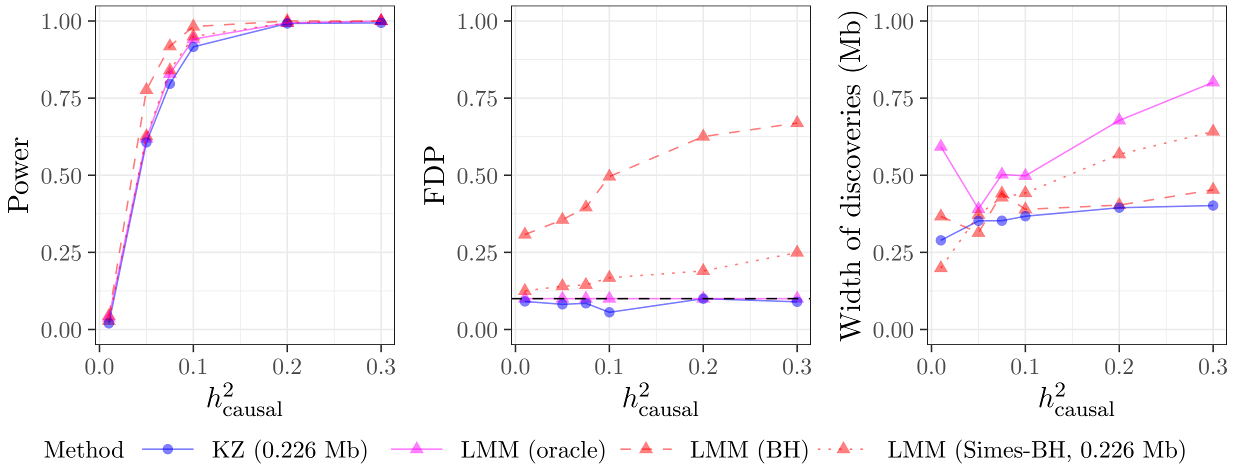

(a)

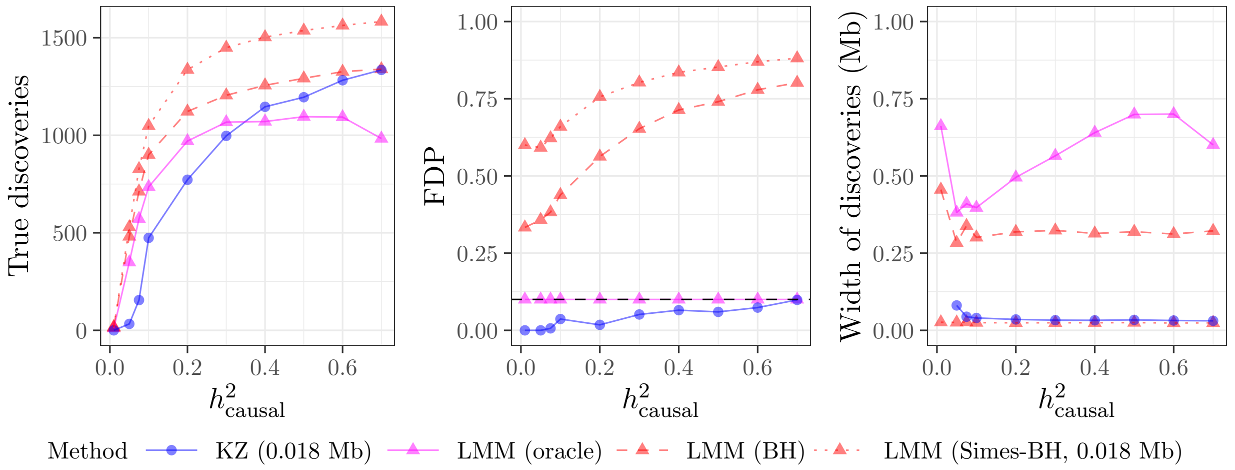

(b)

SUPPLEMENTARY FIGURE 6. **Heuristic FDR control for the LMM.** Performance of *KnockoffZoom* and two alternative LMM-based heuristics for FDR control, for the same experiments as in Figure 3 of the main text. In (b) we do not simplify the *KnockoffZoom* findings at multiple resolutions; instead, we only report those at a fixed resolution, for comparison with the Simes-BH method.

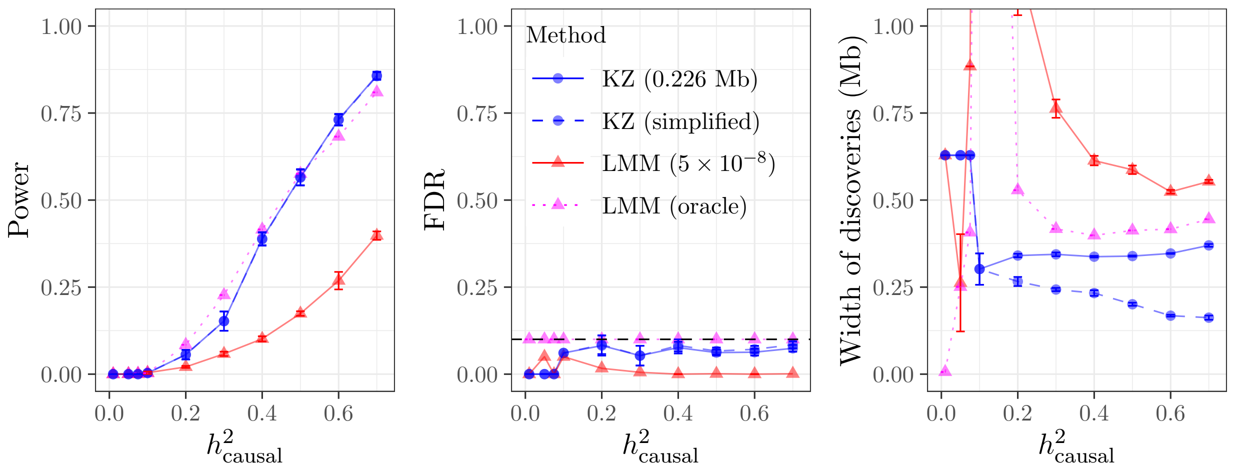

(a)

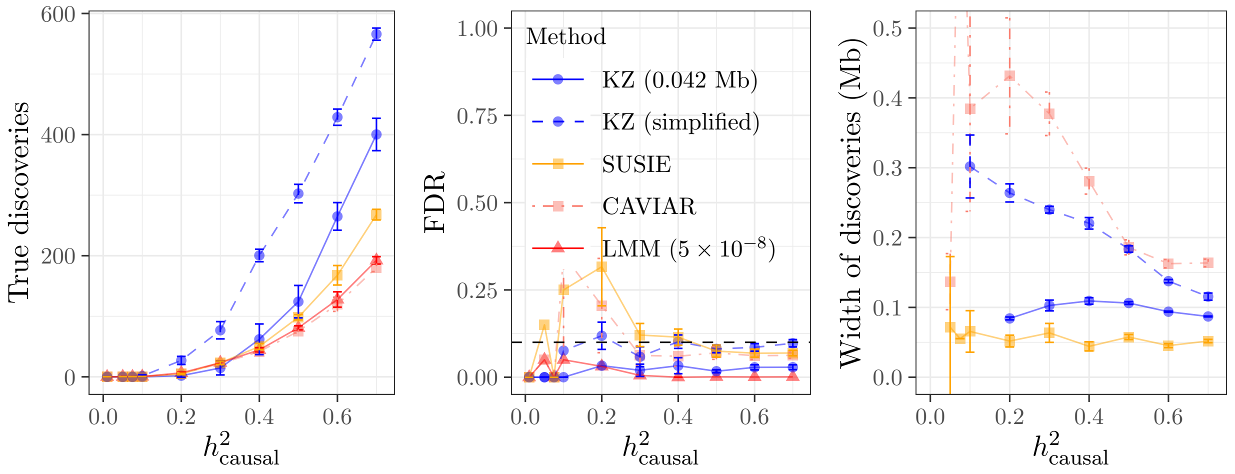

(b)

SUPPLEMENTARY FIGURE 7. **Average performance in repeated simulations.** Average performance of *KnockoffZoom* and BOLT-LMM for simulated phenotypes, repeating the experiments 10 times on disjoint subsets of the data, each including 30k individuals. The error bars indicate 95% confidence intervals for the mean quantities, estimated from the 10 independent experiments. The other details in (a) and (b) are as in Figures 3 and Figure 4 of the main text, respectively.

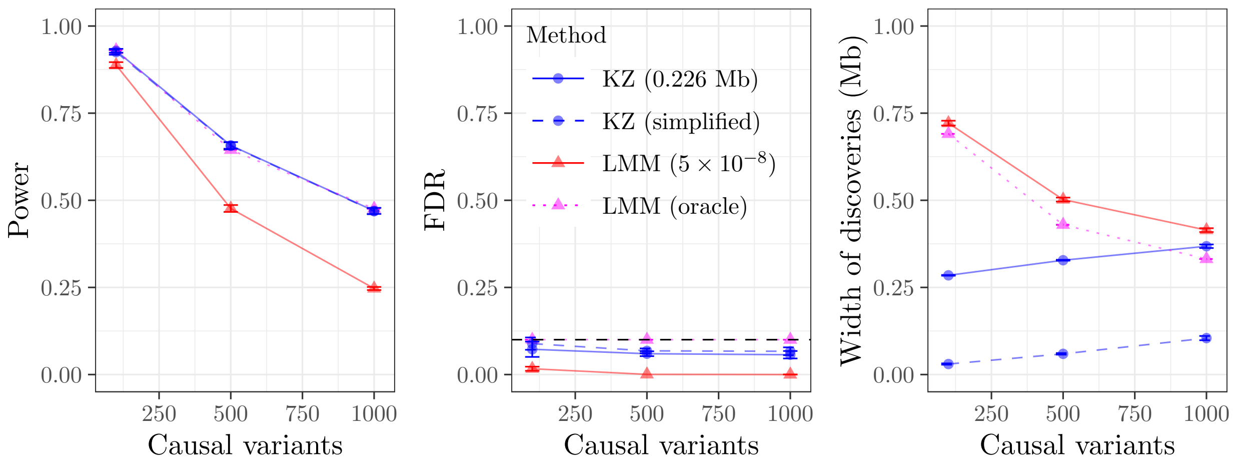

(a)

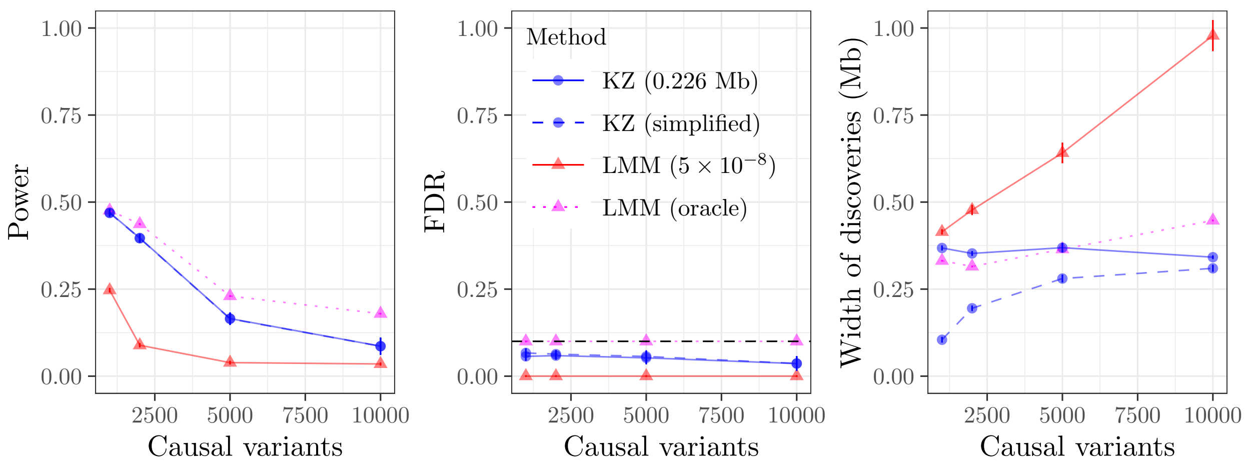

(b)

SUPPLEMENTARY FIGURE 8. **Additional repeated simulations.** Average performance of *KnockoffZoom* and BOLT-LMM for a simulated trait with different genetic architectures from Supplementary Table 3, as a function of the number of causal variants. The heritability of the trait is $h^2_{\text{causal}} = 0.5$. (a): architectures 3,4,5; (b): architectures 5,6,7,8. Other details as in Supplementary Figure 7 (a).

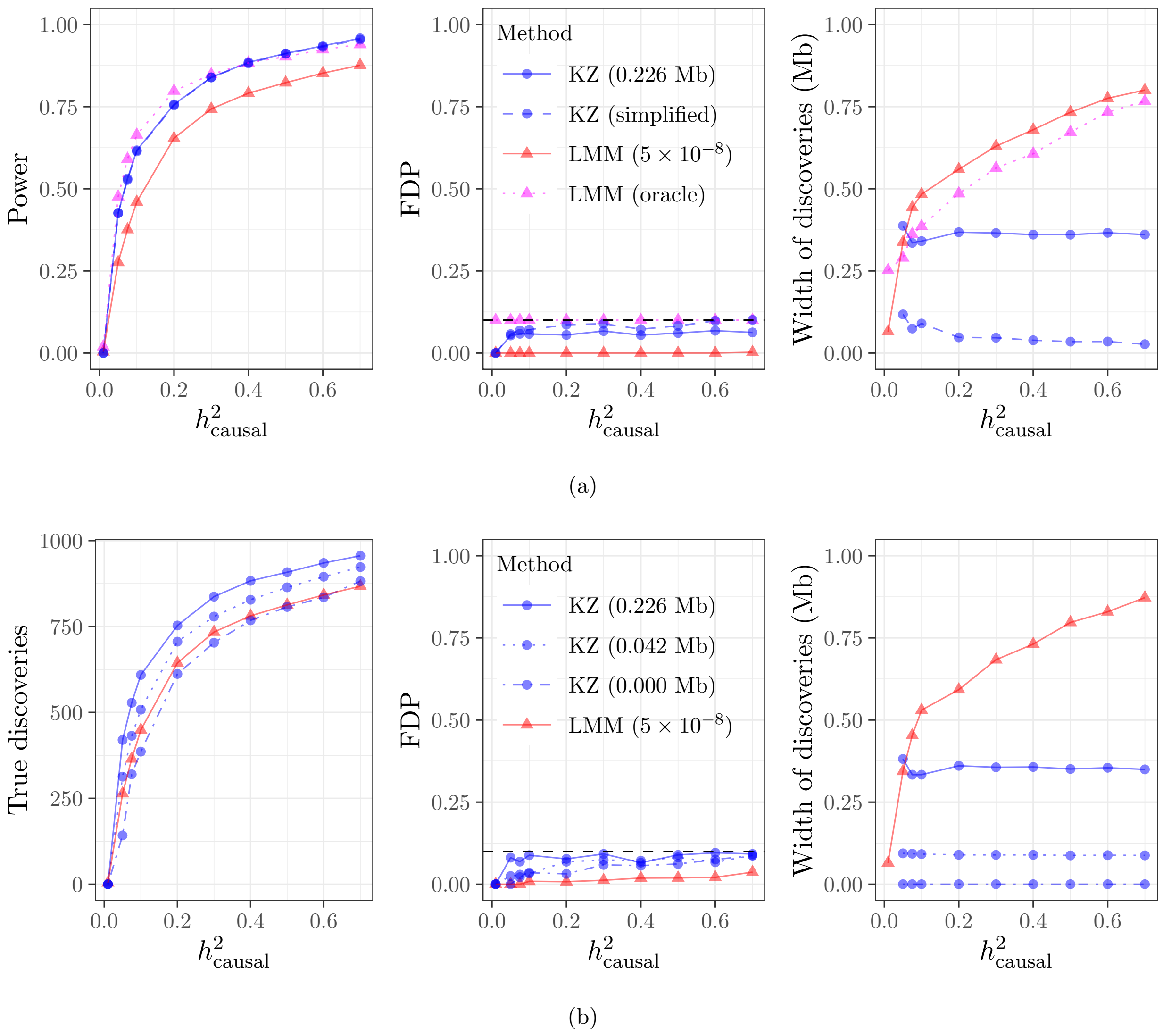

SUPPLEMENTARY FIGURE 9. **Additional locus-discovery simulations.** Performance of *KnockoffZoom* and BOLT-LMM for a simulated trait with genetic architecture 5 from Supplementary Table 3, in which all causal SNPs are well-separated. (b): *KnockoffZoom* at different levels of resolutions. Other details as in Figure 3 of the main text.

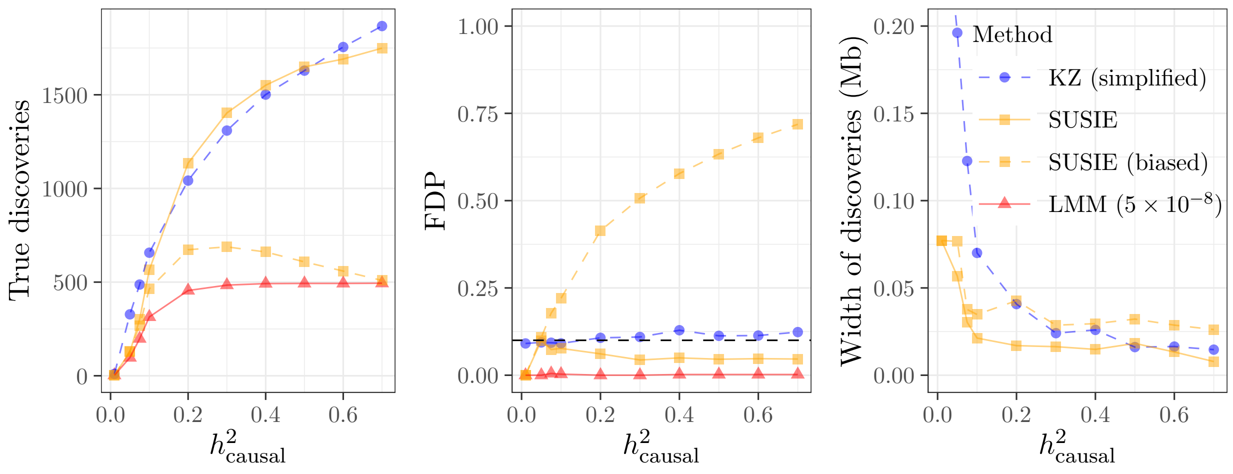

SUPPLEMENTARY FIGURE 10. **Selection bias in two-step fine-mapping.** Fine-mapping with BOLT-LMM followed by SUSIE, with and without mitigating the selection bias. Other details as in Figure 4 of the main text. The biased results refer to SUSIE applied exactly on the clumps reported by BOLT-LMM, without including nearby unselected SNPs.

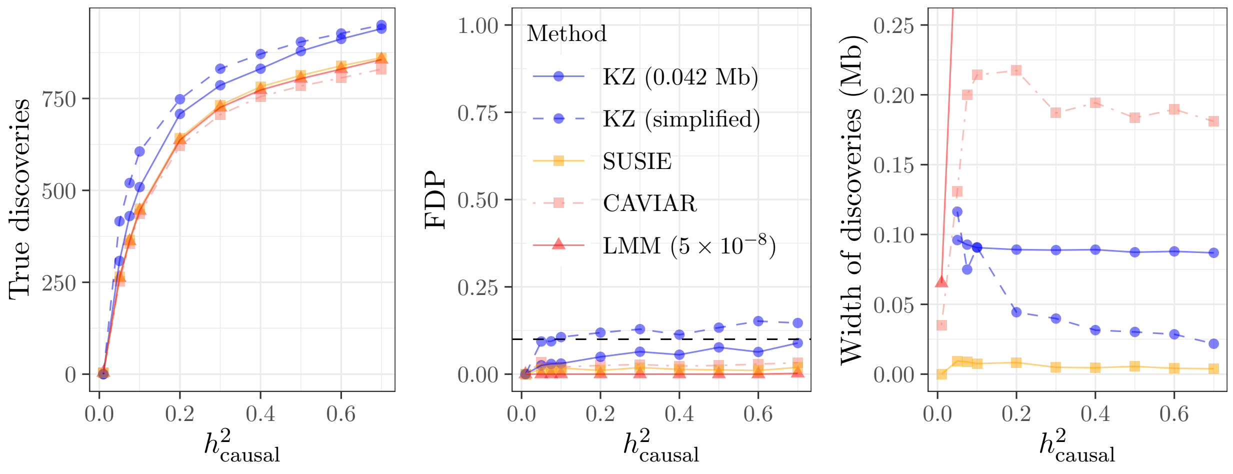

SUPPLEMENTARY FIGURE 11. **Additional fine-mapping simulations.** Fine-mapping performance of *KnockoffZoom* and BOLT-LMM followed by CAVIAR or SUSIE. Simulated trait as in Supplementary Figure 9 (genetic architecture 5 from Supplementary Table 3). Other details as in Figure 4 of the main text. Here, *KnockoffZoom* is more powerful than SUSIE.

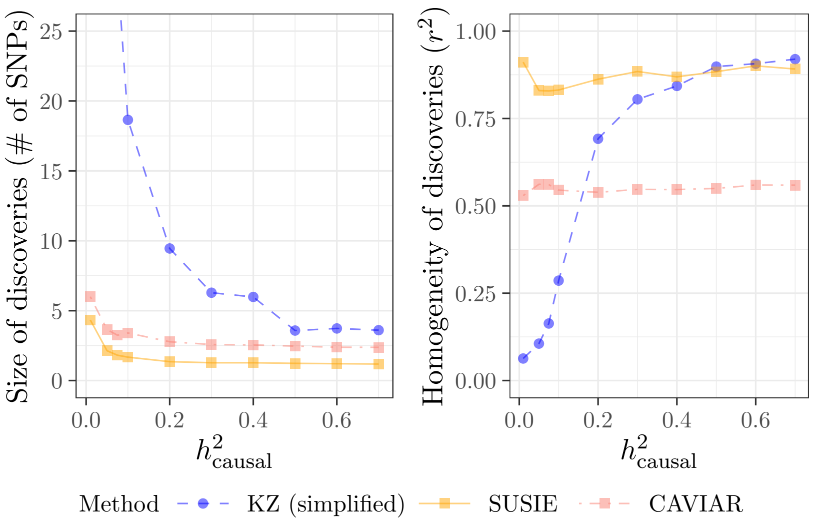

(a)

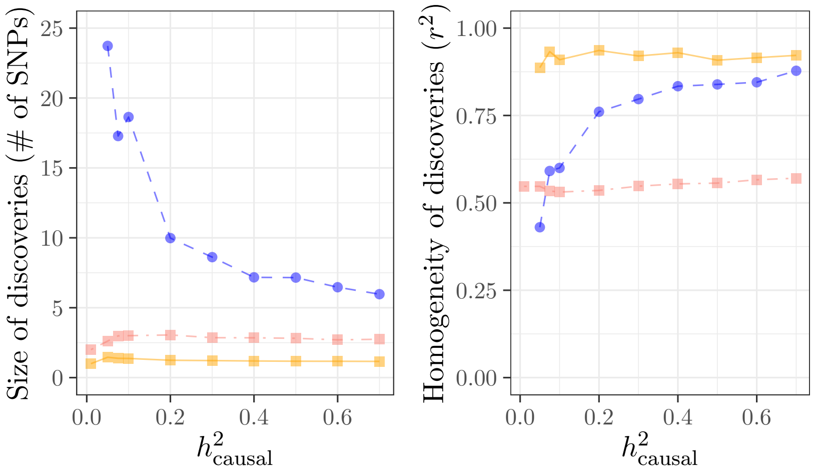

(b)

SUPPLEMENTARY FIGURE 12. **Homogeneity of discoveries in simulations.** Resolution of fine-mapping discoveries in terms of average number of reported SNPs according to different measures, as a function of the heritability of the simulated trait. Left: average width measured in Mb (lower is better); center: average size measured in number of SNPs (lower is better); right: average homogeneity measured in mean pairwise r^2 (higher is better). (a): other details as in Figure 4 of the main text; (b): other details as in Supplementary Figure 11.

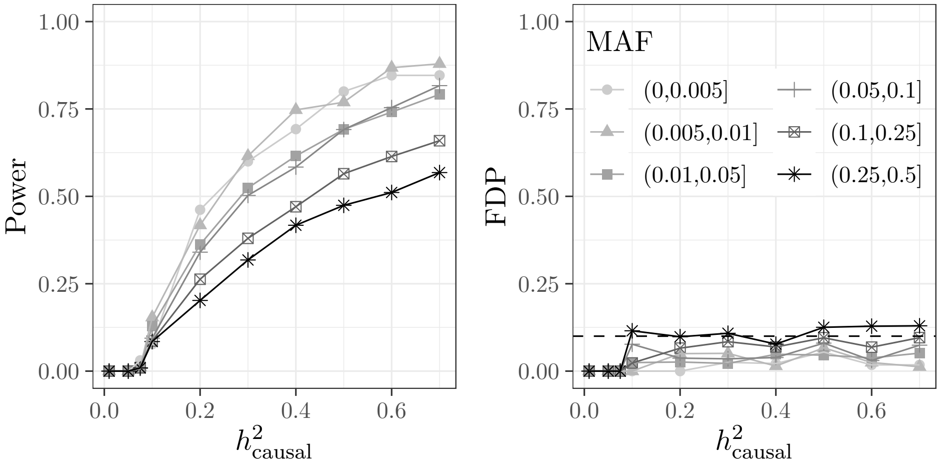

(a)

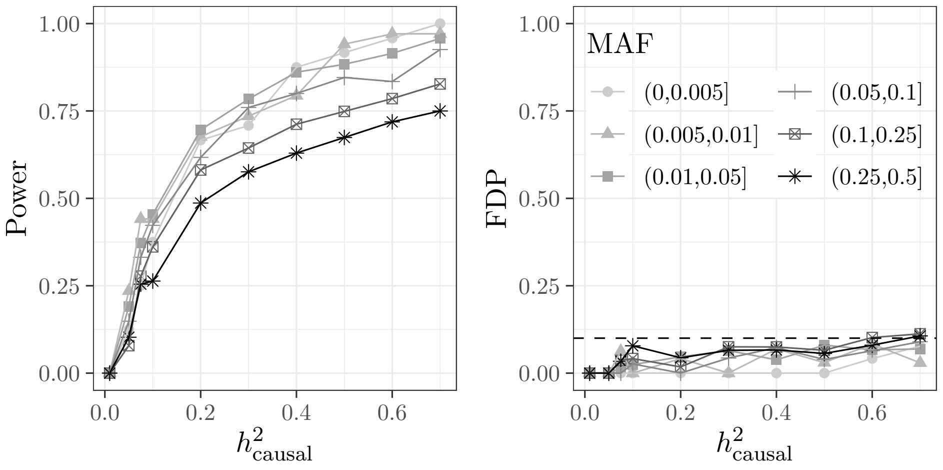

(b)

SUPPLEMENTARY FIGURE 13. **Effect of allele frequency in simulations.** Fine-mapping performance of *KnockoffZoom* at the single-SNP resolution, stratified by the minor allele frequency of the causal variants. (a): other details as in Figure 4 of the main text; (b): other details as in Supplementary Figure 11. This shows that our method performs well both for higher and lower-frequency variants, even though the knockoffs for lower-frequency variants are less accurate (Supplementary Figure 2). Furthermore, *KnockoffZoom* appears to be more powerful for lower-frequency variants in these simulations, which is not very surprising given that there are stronger signals on rarer variants (i.e., the effect sizes are scaled by the inverse standard deviation of the allele count). Although the power will generally depend on the true effect sizes and allele frequencies, it is reassuring to observe that *KnockoffZoom* is not intrinsically limited by lower-frequency variants.

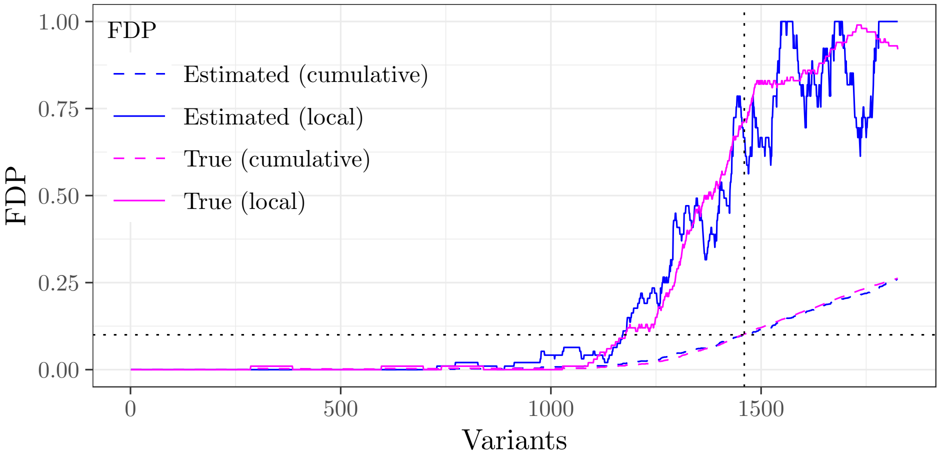

SUPPLEMENTARY FIGURE 14. **Estimated local FDP in simulations.** Estimated and true proportion of false discoveries obtained with *KnockoffZoom* at medium resolution (0.018 Mb), for a simulated trait with 2500 causal variants. The dotted vertical line indicates the adaptive significance threshold computed by the knockoff filter at the nominal FDR significance of 0.1. This corresponds exactly to the last crossing of the 0.1 level (dotted horizontal line) by the estimated cumulative FDP curve. The local (or estimated) FDP is computed by looking at 100 statistics within a rolling window.

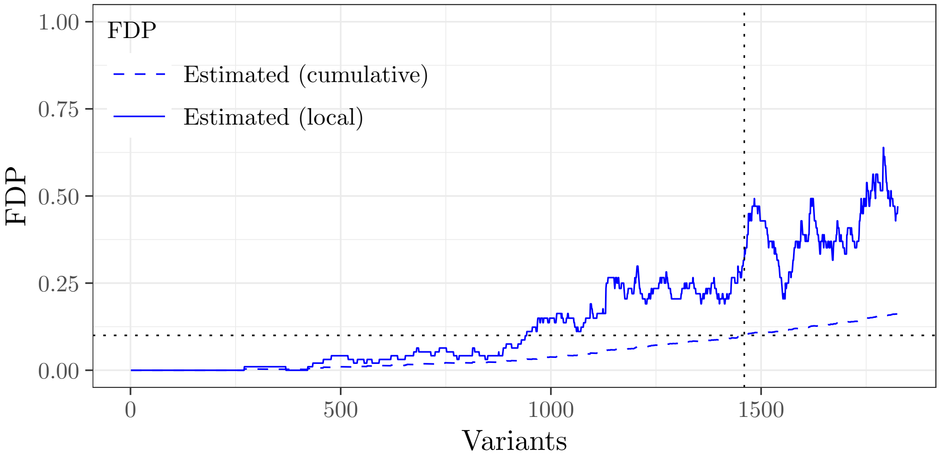

SUPPLEMENTARY FIGURE 15. **Estimated local FDP for real data.** Estimated proportion of false discoveries obtained with *KnockoffZoom* at low-resolution (0.226 Mb) for the phenotype *platelet* in the UK Biobank. Other details as in Supplementary Figure 14.

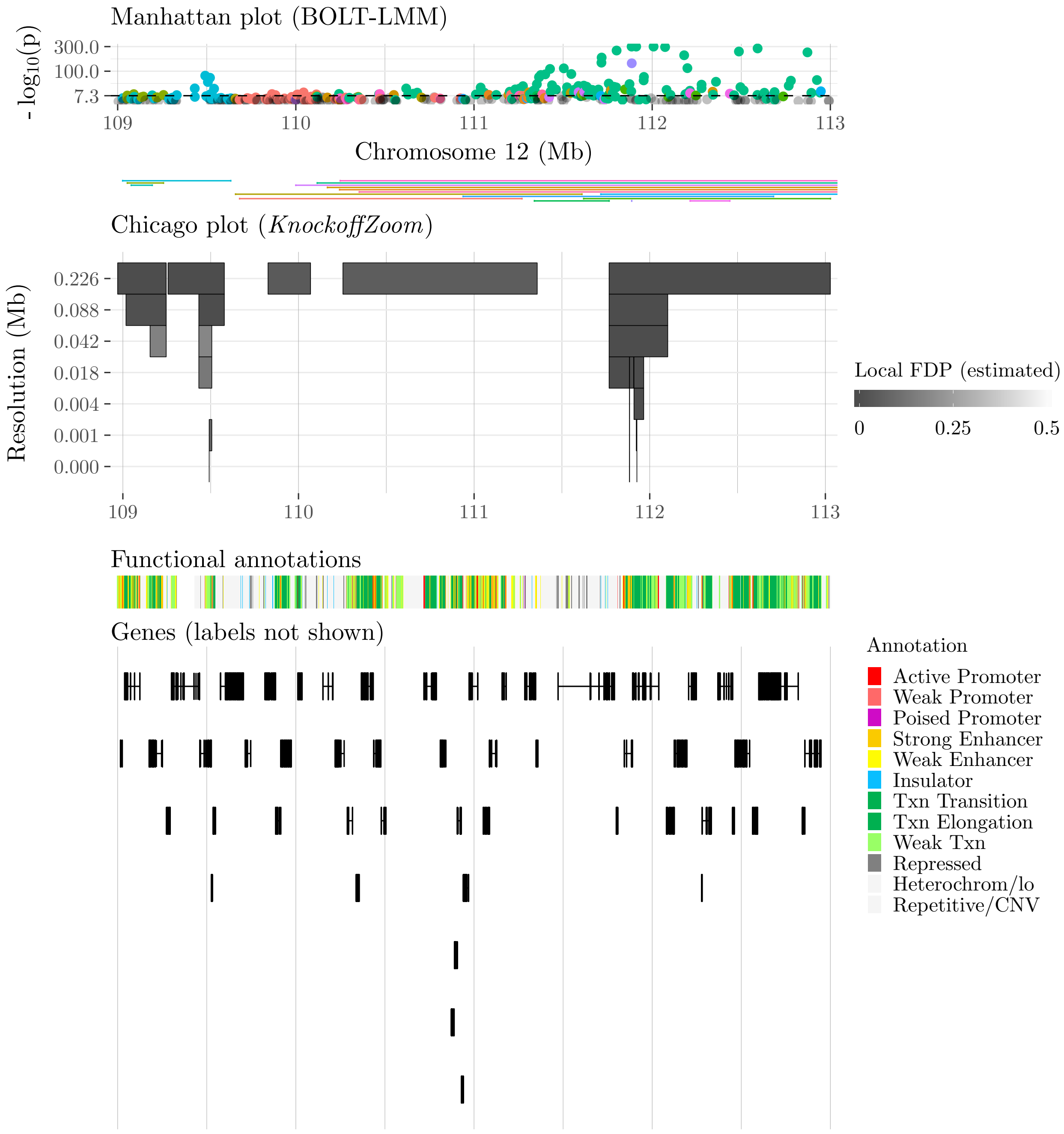

SUPPLEMENTARY FIGURE 16. **Additional visualization of discoveries for platelet count.** Visualization of some discoveries made with *KnockoffZoom* and BOLT-LMM for *platelet* in the UK Biobank. An estimate of the local FDP computed as in Supplementary Figure 15 is included for each level of resolution. Other details as in Figures 1 and 5 of the main text. This Chicago plot is upside-down.

SUPPLEMENTARY TABLES

Resolution	Number of blocks	Mean block width (Mb)	Mean block size (# SNPs)	Mean knockoff r^2
100%	591513	single-SNP	1.00	0.739
75%	443636	0.001	1.33	0.732
50%	295754	0.004	2.00	0.722
20%	118300	0.018	5.00	0.686
10%	59151	0.042	10.00	0.606
5%	29576	0.088	20.00	0.458
2%	11831	0.226	50.0	0.231

SUPPLEMENTARY TABLE 1. **Genome partitions.** Summary of the *KnockoffZoom* partitions of the genome at 7 different levels of resolutions, for 591,513 SNPs in the UK Biobank. The last column indicates the average pairwise r^2 between genotypes and knockoffs at each resolution. Larger values of r^2 at higher resolutions reflect the intrinsic difficulty of fine-mapping. Conversely, smaller values of r^2 at lower resolutions allow more powerful test statistics for locus-discovery. The full distribution of these r^2 coefficients at different resolutions is shown in the boxplots of Supplementary Figure 1.

Module	Operation	Time (h)	Memory (GB)	Machines	Cores
1	convert haplotypes	30	50	22	1
1	fit HMM	144	50	22	1
2	compute LD matrix	2	50	22	1
2	hierarchical clustering	8	200	22	1
3	generate knockoffs	72	30	22	1
3	combine augmented data	8	60	1	1
4	memory mapping	12	60	1	1
4	sparse regression	12	20	1	10

SUPPLEMENTARY TABLE 2. **Computational cost.** Computation time and resources for each module of *KnockoffZoom*, for the analysis of the phenotype *height* in the UK Biobank (591k SNPs and 350k individuals).

Name	Number of causal regions	Causal variants per region	Total number of causal variants	Width of causal regions (Mb)	Figures
1	500	5	2500	0.1	2–4 (main text), 5–7, 10, 12–14
2	500	1	500	0.1	6 (main text)
3	100	1	100	0.1	8
4	100	5	500	0.1	8
5	1000	1	1000	0.1	5, 8, 9, 11–13
6	1000	2	2000	0.1	5, 8
7	1000	5	5000	0.1	5, 8
8	1000	10	10000	0.1	5, 8

SUPPLEMENTARY TABLE 3. **Genetic architectures in simulations.** Genetic architectures used in the simulations. The first architecture is used in the main numerical experiments in the paper, while the simulated case-control study is based on the second one. The other architectures are considered in the additional experiments presented in this supplement. Unless specified otherwise, the references point to Supplementary Figures.

Name	Description	Number of cases	UK Biobank Fields	UK Biobank Codes
height	standing height	continuous	50-0.0	
bmi	body mass index	continuous	21001-0.0	
sbp	systolic blood pressure	continuous	4080-0.0, 4080-0.1	
platelet	platelet count	continuous	30080-0.0	
cvd	cardiovascular disease	116454	20002-0.0–20002-0.32	1065, 1066, 1067, 1068, 1081, 1082, 1083, 1425, 1473, 1493
diabetes	diabetes	14848	20002-0.0–20002-0.32	1220
hypothyroidism	hypothyroidism	17985	20002-0.0–20002-0.32	1226
respiratory	respiratory disease	51625	20002-0.0–20002-0.32	1111, 1112, 1113, 1114, 1115, 1117, 1413, 1414, 1415, 1594
glaucoma	glaucoma	2656	4689-0.0–4689-2.0	age $>$ 30

SUPPLEMENTARY TABLE 4. **Phenotype definitions.** Phenotype definition in the UK Biobank. The numbers of disease cases refer to the subset of unrelated British individuals that passed our quality control.

Phenotype	Resolution													
	0.226 Mb		0.088 Mb		0.042 Mb		0.018 Mb		0.004 Mb		0.001 Mb		single-SNP	
	PC	no PC	PC	no PC	PC	no PC	PC	no PC	PC	no PC	PC	no PC	PC	no PC
height	3284	3252	1976	2007	823	785	388	391	336	335	170	214	173	167
bmi	1804	1808	555	471	60	46	33	33	24	24	0	21	15	17
platelet	1460	1479	890	880	408	413	276	236	161	200	181	156	143	146
sbp	722	745	297	322	95	129	0	0	0	0	0	0	0	0
cvd	514	475	182	164	51	51	0	0	0	0	0	0	0	0
hypothyroidism	212	163	108	103	0	0	0	0	0	0	0	0	21	21
respiratory	176	183	65	60	41	12	13	13	14	14	12	12	0	0
diabetes	50	48	33	33	21	18	10	10	11	12	10	10	0	0
glaucoma	0	0	0	0	0	0	0	0	0	0	0	0	0	0

SUPPLEMENTARY TABLE 5. **Effect of principal components on discoveries.** Number of distinct *KnockoffZoom* findings at different resolutions, with and without including principal components. The findings obtained with principal components correspond to those in Table 1 of the main text.

Phenotype	Method	Discoveries	Overlapping	Distinct
height	*KnockoffZoom*	3284	2246	1038
	BOLT-LMM	1685	1679	6
bmi	*KnockoffZoom*	1804	627	1177
	BOLT-LMM	389	378	11
platelet	*KnockoffZoom*	1460	848	612
	BOLT-LMM	723	709	14
sbp	*KnockoffZoom*	722	243	479
	BOLT-LMM	197	188	9
cvd	*KnockoffZoom*	514	188	326
	BOLT-LMM	156	144	12
hypothyroidism	*KnockoffZoom*	212	96	116
	BOLT-LMM	96	91	5
respiratory	*KnockoffZoom*	176	59	117
	BOLT-LMM	63	59	4
diabetes	*KnockoffZoom*	50	40	10
	BOLT-LMM	47	43	4
glaucoma	*KnockoffZoom*	0	0	0
	BOLT-LMM	5	0	5

SUPPLEMENTARY TABLE 6. **Overlap of *KnockoffZoom* and BOLT-LMM discoveries.** Overlap of the discoveries made with *KnockoffZoom* at low resolution (0.226 Mb) and BOLT-LMM (clumped without consolidation), using the same data. *KnockoffZoom* is applied as in Table 1 of the main text. For example, our method reports 3284 findings for *height*, 2246 of which overlap at least one of the 1685 clumps found by the LMM, while 1038 are distinct from those found by the LMM. Conversely, only 6 out of 1685 discoveries made by BOLT-LMM are not detected by *KnockoffZoom*. We say that findings made by different methods are overlapping if they are within 0.1 Mb of each other. These results confirm that our method makes many new findings, in addition to refining those reported by BOLT-LMM.

Phenotype	*KnockoffZoom* 350k unrelated British			BOLT-LMM 459k European			
		Overlapping with BOLT-LMM		(5×10^{-9})		(5×10^{-8})	
	Discoveries	(5×10^{-9})	(5×10^{-8})	Discoveries	Overlapping with *KnockoffZoom*	Discoveries	Overlapping with *KnockoffZoom*
height	3284	2339	2547	2056	2033	2464	2431
bmi	1804	778	967	504	493	697	672
platelet	1460	1025	1111	1016	988	1204	1155
sbp	722	401	461	440	371	568	452
cvd	514	203	257	192	173	257	229
hypothyroidism	212	111	137	118	112	143	134

SUPPLEMENTARY TABLE 7. **Comparison with BOLT-LMM on a larger sample.** Comparison of the low-resolution (0.226 Mb) discoveries reported by *KnockoffZoom*, as in Table 1 of the main text, and those obtained by BOLT-LMM with a larger dataset of 459,327 European subjects in the UK Biobank.[18]. For example, BOLT-LMM reports 2056 discoveries for *height* at the significance level 5×10^{-9}, 2033 of which overlap with at least one of our 3284 findings, while 2339 of our findings overlap with at least one discovery made by BOLT-LMM.

Phenotype	Resolution																				
	0.226 Mb			0.088 Mb			0.042 Mb			0.018 Mb			0.004 Mb			0.001 Mb			single-SNP		
	no 1.93	1.93		no 1.93	1.93		no 1.93	1.93		no 1.93	1.93		no 1.93	1.93		no 1.93	1.93		no 1.93	1.93	
height	3284	2322	3284	1958	1228	1976	785	335	823	375	101	388	274	23	336	117	0	170	106	0	173
bmi	1804	1249	1804	536	297	555	60	0	60	31	0	33	22	0	24	0	0	0	0	0	15
platelet	1460	1123	1460	870	505	890	393	236	408	227	145	276	136	60	161	119	50	181	100	29	143
sbp	722	506	722	289	183	297	91	42	95	0	0	0	0	0	0	0	0	0	0	0	0
cvd	514	367	514	167	0	182	50	0	51	0	0	0	0	0	0	0	0	0	0	0	0
hypothyroidism	212	141	212	108	0	108	0	0	0	0	0	0	0	0	0	0	0	0	0	0	21
respiratory	176	119	176	63	0	65	11	0	41	0	0	13	0	0	14	0	0	12	0	0	0
diabetes	50	44	50	33	30	33	19	0	21	10	0	10	0	0	11	0	0	10	0	0	0
glaucoma	0	0	0	0	0	0	0	0	0	0	0	0	0	0	0	0	0	0	0	0	0

SUPPLEMENTARY TABLE 8. **Coordinated multi-resolution discoveries.** Distinct findings made by *KnockoffZoom* using the consistent-layers knockoff filter, without or with the 1.93 correction factor. The third number in each cell (on the new line) indicates the number of discoveries obtained separately at each resolution, without applying the consistent-layers knockoff filter.

	Min.	1st Qu.	Median	Mean	3rd Qu.	Max.
All variants	0.001	0.025	0.066	0.133	0.213	0.500
Discoveries						
height	0.001	0.091	0.224	0.230	0.360	0.500
bmi	0.001	0.112	0.244	0.241	0.364	0.500
platelet	0.001	0.091	0.225	0.230	0.355	0.500
sbp	0.001	0.098	0.238	0.239	0.371	0.500
cvd	0.002	0.114	0.248	0.246	0.368	0.496
hypothyroidism	0.001	0.121	0.246	0.247	0.373	0.497
respiratory	0.001	0.082	0.234	0.228	0.354	0.496
diabetes	0.007	0.128	0.279	0.259	0.382	0.494

SUPPLEMENTARY TABLE 9. **Effect of allele frequency on discoveries.** Minor allele frequency distribution of the lead variants selected by *KnockoffZoom* at low resolution (0.226 Mb) for different traits, compared to the distribution of all genotyped variants in the UK Biobank. The lead variant in each selected group is defined as that having the largest estimated regression coefficient in absolute value. These results show that lower-frequency variants are less likely to be selected by *KnockoffZoom*. This observation is consistent with the fact that effects on rarer variants are intrinsically harder to detect.

Phenotype	Method	# Discoveries		
		Total	Overlapping	Distinct
height	*KnockoffZoom*	121	56	65
	BOLT-LMM	54	49	5
platelet	*KnockoffZoom*	81	44	37
	BOLT-LMM	47	42	5

SUPPLEMENTARY TABLE 10. **Overlap of discoveries in reproducibility study.** Overlap of the discoveries made with *KnockoffZoom* (low-resolution) and BOLT-LMM (clumped without consolidation). Other details as in Table 2 of the main text. For example, we make 121 discoveries for *height*, 56 of which are within 0.1 Mb of at least one of the 54 clumps found by the LMM, while 65 are distinct. In this case, only 5 out of 54 discoveries made by BOLT-LMM are not detected by *KnockoffZoom*.

		30k unrelated British			459k European (BOLT-LMM)			
		Discoveries			Discoveries		Discoveries (consolidated)	
Phenotype	Method	#	Not replicated	Size (Mb)	Anticipated	Size (Mb)	Anticipated	Size (Mb)
height	*KnockoffZoom*	121	8 (6.6%)	0.308	398/2056 (19.4%)	0.789	92/807 (11.4%)	1.162
	LMM	54	0 (0.0%)	0.965	280/2056 (13.6%)	0.789	48/807 (5.9%)	1.162
	LMM (BH)	714	203 (28.4%)	0.379	1047/2056 (50.9%)	0.789	325/807 (40.3%)	1.162
platelet	*KnockoffZoom*	81	5 (6.2%)	0.319	235/1016 (23.1%)	0.805	65/525 (12.4%)	0.953
	LMM	47	0 (0.0%)	0.674	219/1016 (21.6%)	0.805	42/525 (8.0%)	0.953
	LMM (BH)	272	92 (33.8%)	0.433	422/1016 (41.5%)	0.805	136/525 (25.9%)	0.953

SUPPLEMENTARY TABLE 11. **Relative power in reproducibility study.** Estimated low-resolution power of *KnockoffZoom* and BOLT-LMM. Other details as in Supplementary Table 10. We say that a finding reported by BOLT-LMM on the larger dataset is anticipated by those in the smaller dataset if it is within 0.1 Mb of at least one of them. For example, among the 2056 unconsolidated discoveries reported by BOLT-LMM for *height* on the large dataset, 398 are anticipated by the findings of *KnockoffZoom* on the smaller dataset, while only 280 are anticipated by BOLT-LMM using the same data.

Resolution	Platelet activation	Hemostasis	Blood coagulation	Wound healing	Response to wounding
0.226	1.8×10^{-08}	2.8×10^{-12}	3.9×10^{-12}	2.0×10^{-11}	5.6×10^{-13}
0.088	2.8×10^{-10}	3.1×10^{-20}	6.1×10^{-20}	1.2×10^{-19}	4.8×10^{-21}
0.042	1.0×10^{-08}	1.1×10^{-20}	3.6×10^{-20}	3.6×10^{-18}	6.8×10^{-17}
0.018	5.4×10^{-08}	3.0×10^{-21}	1.3×10^{-20}	4.0×10^{-18}	3.9×10^{-17}
0.004	2.8×10^{-09}	5.4×10^{-23}	3.4×10^{-22}	1.8×10^{-19}	4.7×10^{-17}
0.001	3.5×10^{-09}	3.0×10^{-23}	1.8×10^{-22}	3.1×10^{-20}	3.5×10^{-19}
single-SNP	2.6×10^{-09}	1.8×10^{-24}	1.4×10^{-23}	5.4×10^{-21}	1.8×10^{-17}

SUPPLEMENTARY TABLE 12. **Enrichment analysis.** Gene ontology enrichment analysis using GREAT[29] for the *KnockoffZoom* results on *platelet*. The uncorrected p-values for 5 relevant biological processes are shown at each resolution.

Consequence	Discoveries
nonsense	1
missense	26
synonymous	2
3-prime UTR	7
splice donor	1
intronic	74
500B downstream	2
2KB upstream	4
intergenic	26

SUPPLEMENTARY TABLE 13. **Discovered variants with known consequences.** Most serious previously known consequence (dbSNP[30]) of each of the 143 variants discovered by *KnockoffZoom* at the highest resolution for the phenotype *platelet* in the UK Biobank.

Previous association	Discoveries
platelet	61
hemoglobin	4
red cells	2
squamous cell carcinoma	2
white cells	2
breast cancer	1
coronary artery disease	1
fatty liver disease	1
macular degeneration	1
menarche (age of onset)	1
obesity	1
none	66

SUPPLEMENTARY TABLE 14. **Known associations of high-resolution discoveries.** Most relevant previously reported associations for some of the 143 variants discovered by *KnockoffZoom* at the highest resolution for the phenotype *platelet* in the UK Biobank.

Chromosome	SNP	Position	Gene	Consequence
19	Affx-15656246	19765499	ATP13A1	missense
19	rs12983010	39229089	CAPN12	missense
1	rs140584594	110232983	GSTM1	missense
17	rs79007502	33880305	SLFN14	missense
2	rs76774368	160604514	MARCH7	missense
3	rs34095724	184099050	CHRD	missense

SUPPLEMENTARY TABLE 15. **New missense discoveries.** Missense variants localized by *Knockoff-Zoom* at the highest resolution, for the phenotype *platelet* in the UK Biobank, that have not been reported before.

Resolution	Phenotype	Seed 1		Seed 2		Both seeds		Stable (%)	
		no CL	CL	no CL	CL	no CL	CL	no CL	CL
0.226 Mb	height	3284		3155		2792		86.8	
	bmi	1804		1841		1508		82.8	
	platelet	1460		1424		1196		83.0	
	sbp	722		802		616		81.1	
	cvd	514		472		379		77.0	
	hypothyroidism	212		186		167		84.3	
	respiratory	176		158		131		78.7	
0.088 Mb	height	1976	1958	1803	1791	1577	1562	83.6	83.5
	bmi	555	536	670	646	447	426	73.6	72.7
	platelet	891	870	975	914	755	729	81.1	81.8
	sbp	297	289	360	323	234	223	71.9	73.1
	cvd	182	167	227	215	147	131	72.8	69.7
	hypothyroidism	108	108	77	76	67	67	74.5	75.1
0.042 Mb	height	823	785	841	810	655	625	78.7	78.4
	platelet	408	393	466	442	326	310	74.9	74.5
0.018 Mb	height	388	375	331	304	265	244	74.2	72.7
	platelet	276	227	231	213	185	164	73.6	74.6
0.004 Mb	height	336	274	202	145	175	127	69.4	67.0
	platelet	161	136	226	180	139	112	73.9	72.3
0.001 Mb	height	170	117	121	103	100	78	70.7	71.2
	platelet	181	119	202	154	148	97	77.5	72.2
single-SNP	height	173	106	134	92	115	72	76.1	73.1
	platelet	143	100	124	100	100	72	75.3	72.0

SUPPLEMENTARY TABLE 16. **Variability of *KnockoffZoom* discoveries.** Numbers of *KnockoffZoom* discoveries obtained with different random seeds for the generation of knockoffs. The first and second values in each column refer to the results obtained without and with explicit coordination of results at different resolutions using the consistent-layers knockoff filter (these are equivalent at the lowest resolution). The stability percentage is defined as the average proportion of *KnockoffZoom* discoveries that are consistently reported with both random seeds. Only the analyses for which *KnockoffZoom* reports at least 100 discoveries with the first random seed are shown here. These results show that the *KnockoffZoom* results are quite stable (approximately 80% of the discoveries obtained with two different random seeds match), especially when the number of discoveries is large. In fact, most of the variability affects signals that are close to the FDR significance level and hence most difficult to detect (perhaps because they are tested at very high resolution, or because they have weak signals, or because they are false positives). We can see this explicitly in Supplementary Tables 17–20, where the discoveries are stratified by their individual significance measured in terms of the estimated local FDP.

Phenotype	Estimated local FDP ≤ 0.1				Estimated local FDP > 0.1			
	Seed 1	Seed 2	Both	Stable (%)	Seed 1	Seed 2	Both	Stable (%)
height	1886	1870	1752	93.3	1398	1285	1040	77.7
bmi	1080	1091	984	90.7	724	750	524	71.1
platelet	932	927	836	89.9	528	497	360	70.3
sbp	456	490	421	89.1	266	312	195	67.9
cvd	356	332	289	84.1	158	140	90	60.6
hypothyroidism	162	158	143	89.4	50	28	24	66.9
respiratory	160	154	129	82.2	16	4	2	31.2

SUPPLEMENTARY TABLE 17. **Low-resolution variability and local FDP.** Numbers of *KnockoffZoom* discoveries at low resolution (0.226 Mb) using different random seeds. These discoveries are stratified by their estimated local FDP. These results indicate that discoveries with lower local FDP (those about which we are more confident) are much more stable.

Phenotype	Estimated local FDP ≤ 0.1				Estimated local FDP > 0.1			
	Seed 1	Seed 2	Both	Stable (%)	Seed 1	Seed 2	Both	Stable (%)
height	1033	988	907	89.8	943	815	670	76.6
bmi	335	432	289	76.6	220	238	158	69.1
platelet	519	537	469	88.9	372	438	286	71.1
sbp	220	240	184	80.2	77	120	50	53.3
cvd	174	200	140	75.2	8	27	7	56.7

SUPPLEMENTARY TABLE 18. **Mid-resolution variability and local FDP.** Numbers of *KnockoffZoom* discoveries at intermediate resolution (0.088 Mb) using different random seeds. Other details as in Supplementary Table 17.

Phenotype	Estimated local FDP ≤ 0.1				Estimated local FDP > 0.1			
	Seed 1	Seed 2	Both	Stable (%)	Seed 1	Seed 2	Both	Stable (%)
height	153	123	111	81.4	20	11	4	28.2
platelet	128	114	93	77.1	15	10	7	58.3

SUPPLEMENTARY TABLE 19. **High-resolution variability and local FDP.** Numbers of *KnockoffZoom* discoveries at high resolution (single SNP) using different random seeds. Other details as in Supplementary Table 17.

FDP	Seed 1			Seed 2		
	Discoveries	Confirmed	Stable (%)	Discoveries	Confirmed	Stable (%)
[0,0.01)	553	529	95.7	679	644	94.8
[0.01,0.02)	343	323	94.2	159	153	96.2
[0.02,0.05)	554	515	93.0	713	667	93.5
[0.05,0.1)	496	445	89.7	359	328	91.4
[0.1,0.2)	753	622	82.6	647	561	86.7
[0.2,0.5)	585	358	61.2	598	439	73.4

SUPPLEMENTARY TABLE 20. **Variability stratified by local FDP.** Variability in the numbers of *KnockoffZoom* discoveries for the phenotype *height* at low resolution (0.226 Mb) using different random seeds, as a function of the estimated local FDP. For example, 96.7% of the discoveries obtained with the first seed whose estimated local FDP is below 0.01 are also found using the second random seed.

Phenotype	BOLT-LMM (5×10^{-8})	*KnockoffZoom*		
		Seed 1	Seed 2	Both seeds
height	2464	2431	2434	2431 (98.7%)
bmi	697	672	672	672 (96.4%)
platelet	1204	1155	1154	1154 (95.8%)
sbp	568	452	482	452 (79.6%)
cvd	257	229	227	227 (88.3%)
hypothyroidism	143	134	133	133 (93.0%)

SUPPLEMENTARY TABLE 21. **Variability and power comparison with BOLT-LMM.** Numbers of discoveries made by BOLT-LMM (459k European individuals) that are reproduced by *KnockoffZoom* (350k unrelated British individuals) at low resolution (0.226 Mb), using different random seeds for the generation of knockoffs. For example, 2431 out of 2464 discoveries reported by BOLT-LMM for *height* are consistently found by *KnockoffZoom* with both random seeds. The *KnockoffZoom* results obtained with the first random seed correspond to those in Supplementary Table 7.

K	1	2	5	10	15	20	30	50	75	100
Imputation error (%)	14.59	10.23	6.74	5.57	5.04	4.77	4.4	4.01	3.75	3.71

SUPPLEMENTARY TABLE 22. **HMM tuning.** Out-of-sample imputation error of our HMM for haplotypes missing at random, as a function of the number K of motifs in the model.

[1] Katsevich, E. & Sabatti, C. Multilayer knockoff filter: controlled variable selection at multiple resolutions. *Ann. Appl. Stat.* **13**, 1–33 (2019).

[2] Sesia, M., Sabatti, C. & Candès, E. J. Gene hunting with hidden Markov model knockoffs. *Biometrika* **106**, 1–18 (2019).

[3] Scheet, P. & Stephens, M. A fast and flexible statistical model for large-scale population genotype data: applications to inferring missing genotypes and haplotypic phase. *Am. J. Hum. Genet.* **78**, 629–644 (2006).

[4] Marchini, J. & Howie, B. Genotype imputation for genome-wide association studies. *Nat. Rev. Genet.* **11**, 499–511 (2010).

[5] O'Connell, J. *et al.* Haplotype estimation for biobank scale datasets. *Nat. Genet.* **48**, 817–820 (2016).

[6] Fearnhead, P. & Donnelly, P. Estimating recombination rates from population genetic data. *Genetics* **159**, 1299–1318 (2001).

[7] Abraham, G., Kowalczyk, A., Zobel, J. & Inouye, M. Performance and robustness of penalized and unpenalized methods for genetic prediction of complex human disease. *Genet. Epidemiol.* **37**, 184–195 (2013).

[8] Vilhjá, B. J., lmsson *et al.* Modeling linkage disequilibrium increases accuracy of polygenic risk scores. *Am. J. Hum. Genet.* **97**, 576–592 (2015).

[9] Wei, Z. *et al.* Large sample size, wide variant spectrum, and advanced machine-learning technique boost risk prediction for inflammatory bowel disease. *Am. J. Hum. Genet.* **92**, 1008–1012 (2013).

[10] Botta, V., Louppe, G., Geurts, P. & Wehenkel, L. Exploiting SNP correlations within random forest for genome-wide association studies. *PLoS One* **9**, 1–11 (2014).

[11] Bellot, P. & Pérez-Enciso, M. Can deep learning improve genomic prediction of complex human traits? *Genetics* **210**, 809–819 (2018).

[12] Klasen, J. R. *et al.* A multi-marker association method for genome-wide association studies without the need for population structure correction. *Nat. Commun.* **7** (2016).

[13] Privé, F., Aschard, H., Ziyatdinov, A. & Blum, M. G. B. Efficient analysis of large-scale genome-wide data with two R, packages: bigstatsr and bigsnpr. *Bioinformatics* **34**, 2781–2787 (2018).

[14] Zeng, Y. & Breheny, P. The biglasso package: A memory-and computation-efficient solver for lasso model fitting with big data in R (2017). Preprint at `https://arxiv.org/abs/1701.05936`.

[15] Candès, E. J., Fan, Y., Janson, L. & Lv, J. Panning for gold: Model-x knockoffs for high-dimensional controlled variable selection. *J. R. Stat. Soc. B.* **80**, 551–577 (2018).

[16] Barber, R. F. & Candès, E. J. Controlling the false discovery rate via knockoffs. *Ann. Stat.* **43**, 2055–2085 (2015).

[17] Efron, B. *Large-Scale Inference: Empirical Bayes Methods for Estimation, Testing, and Prediction* (Cambridge University Press, 2010).

[18] Loh, P.-R., Kichaev, G., Gazal, S., Schoech, A. P. & Price, A. L. Mixed-model association for biobank-scale datasets. *Nat. Genet.* **50**, 906–908 (2018).

[19] Benner, C. *et al.* FINEMAP: efficient variable selection using summary data from genome-wide association studies. *Bioinformatics* **32**, 1493–1501 (2016).

[20] Kichaev, G. *et al.* Integrating functional data to prioritize causal variants in statistical fine-mapping studies. *PLoS Genet.* **10**, 1–16 (2014).

[21] Schaid, D. J., Chen, W. & Larson, N. B. From genome-wide associations to candidate causal variants by statistical fine-mapping. *Nat. Rev. Genet.* **19**, 491–504 (2018).

[22] Wang, G., Sarkar, A. K., Carbonetto, P. & Stephens, M. A simple new approach to variable selection in regression, with application to genetic fine-mapping (2018). Preprint at `https://doi.org/10.1101/501114`.

[23] Hormozdiari, F., Kostem, E., Kang, E. Y., Pasaniuc, B. & Eskin, E. Identifying causal variants at loci with multiple signals of association. *Genetics* **198**, 497–508 (2014).

[24] Benjamini, Y. & Hochberg, Y. Controlling the false discovery rate: a practical and powerful approach to multiple testing. *J. R. Stat. Soc. B.* **57**, 289–300 (1995).

[25] Simes, R. J. An improved Bonferroni procedure for multiple tests of significance. *Biometrika* **73**, 751–754 (1986).

[26] Katsevich, E., Sabatti, C. & Bogomolov, M. Controlling FDR while highlighting distinct discoveries (2018). Preprint at `https://arxiv.org/abs/1809.01792`.

[27] Siegmund, D. O., Zhang, N. R. & Yakir, B. False discovery rate for scanning statistics. *Biometrika* **98**, 979–985 (2011).

[28] Brzyski, D. *et al.* Controlling the rate of GWAS false discoveries. *Genetics* **205**, 61–75 (2017).

[29] McLean, C. Y. *et al.* GREAT improves functional interpretation of cis-regulatory regions. *Nat. Biotech.* **28**, 495–501 (2010).

[30] Smigielski, E. M., Sirotkin, K., Ward, M. & Sherry, S. T. dbSNP: a database of single nucleotide polymorphisms. *Nucleic Acids Research* **28**, 352–355 (2000).
